# Changes in Biomarkers of Tobacco Exposure among Cigarette Smokers Transitioning to ENDS Use: The Population Assessment of Tobacco and Health Study, 2013–2015

**DOI:** 10.3390/ijerph19031462

**Published:** 2022-01-27

**Authors:** Gabriella M. Anic, Brian L. Rostron, Hoda T. Hammad, Dana M. van Bemmel, Arseima Y. Del Valle-Pinero, Carol H. Christensen, Gladys Erives, Lisa M. Faulcon, Benjamin C. Blount, Yuesong Wang, Lanqing Wang, Deepak Bhandari, Antonia M. Calafat, Heather L. Kimmel, Colm D. Everard, Wilson M. Compton, Kathryn C. Edwards, Maciej L. Goniewicz, Binnian Wei, Andrew Hyland, Dorothy K. Hatsukami, Stephen S. Hecht, Raymond S. Niaura, Nicolette Borek, Bridget K. Ambrose, Cindy M. Chang

**Affiliations:** 1 Office of Science, Center for Tobacco Products, Food and Drug Administration, Silver Spring, MD 20993, USA; gabriella.anic@fda.hhs.gov (G.M.A.); hoda.hammad@fda.hhs.gov (H.T.H.); dana.vanbemmel@fda.hhs.gov (D.M.v.B.); arseima.reissig@fda.hhs.gov (A.Y.D.V.-P.); carol.christensen@fda.hhs.gov (C.H.C.); gladys.erives@fda.hhs.gov (G.E.); lisa.faulcon@fda.hhs.gov (L.M.F.); nicolette.borek@fda.hhs.gov (N.B.); bridget.ambrose@fda.hhs.gov (B.K.A.); cindy.chang@fda.hhs.gov (C.M.C.); 2 Division of Laboratory Sciences, Centers for Disease Control and Prevention, Atlanta, GA 30341, USA; bkb3@cdc.gov (B.C.B.); uxi5@cdc.gov (Y.W.); lfw3@cdc.gov (L.W.); xwo1@cdc.gov (D.B.); aic7@cdc.gov (A.M.C.); 3 National Institute on Drug Abuse, National Institutes of Health, Bethesda, MD 20892, USA; heather.kimmel@nih.gov (H.L.K.); colm.everard@nih.gov (C.D.E.); wcompton@nida.nih.gov (W.M.C.); 4 Behavioral Health and Health Policy, Westat, Rockville, MD 20850, USA; katyedwards@westat.com; 5 Department of Health Behavior, Roswell Park Comprehensive Cancer Center, Buffalo, NY 14263, USA; maciej.goniewicz@roswellpark.org (M.L.G.); binnian.wei@roswellpark.org (B.W.); andrew.hyland@roswellpark.org (A.H.); 6 Masonic Cancer Center, University of Minnesota, Minneapolis, MN 55455, USA; hatsu001@umn.edu (D.K.H.); hecht002@umn.edu (S.S.H.); 7 College of Global Public Health, New York University, New York, NY 10003, USA; rn54@nyu.edu

**Keywords:** tobacco, cigarettes, e-cigarettes, electronic nicotine delivery systems (ENDS), biomarkers

## Abstract

Limited data are available for how biomarkers of tobacco exposure (BOE) change when cigarette smokers transition to using electronic nicotine delivery systems (ENDS). Using biomarker data from Waves 1 (2013–2014) and 2 (2014–2015) of the PATH Study, we examined how mean BOE concentrations, including metabolites of nicotine, tobacco-specific nitrosamines (TSNA), polycyclic aromatic hydrocarbons (PAH), and volatile organic compounds (VOC) and metals, changed when 2475 adult smokers transitioned to using ENDS or quit tobacco products. Exclusive smokers who transitioned to dual use had a significant decrease in NNAL (4-(methylnitrosamino)-1-(3-pyridyl)-1-butanol), but not nicotine metabolites, most PAHs, metals, or VOCs. Exclusive smokers who became dual users had significant reductions in total nicotine equivalents, NNAL, and 2CyEMA (acrylonitrile metabolite), but only in those who reduced cigarettes per day (CPD) by >=50%. Smokers who transitioned to exclusive ENDS use had significant reductions in most TSNAs, PAHs, and VOCs; however, nicotine metabolites did not decrease in dual users who became exclusive ENDS users. Smokers who quit tobacco use had significant decreases in nicotine metabolites, all TSNAs, most PAHs, and most VOCs. Cigarette smokers who became dual users did not experience significant reductions in most BOEs. Reductions were impacted by changes in CPD. However, transitioning from smoking to no tobacco or exclusive ENDS use was associated with reduced exposure to most BOEs measured. Future analyses could incorporate additional waves of PATH data and examine changes in biomarker exposure by ENDS device type and CPD.

## 1. Introduction

Biomarkers of tobacco exposure (BOE) are used to characterize human exposure to harmful and potentially harmful constituents (HPHC) resulting from tobacco product use. HPHCs include nicotine, nicotine metabolites, tobacco-specific nitrosamines (TSNA), polycyclic aromatic hydrocarbons (PAH), volatile organic compounds (VOC), and toxic metals [1]. While cigarettes are known to contain relatively high levels of HPHCs, less is known about exposure to these harmful chemicals from the use of electronic nicotine delivery systems (ENDS) and whether dual use of cigarettes and ENDS can reduce exposure to these toxicants. 

E-cigarette aerosol contains many of the HPHCs found in combusted tobacco products including carbonyl compounds, VOC, and TSNA, although at lower levels than in cigarette smoke [2]. Several cross-sectional studies have compared biomarkers of exposure (BOE) to tobacco product toxicants between cigarette smokers and ENDS users [3,4,5,6,7,8,9,10,11]. Those studies observed significantly lower levels of various TSNA, VOC, and some PAH in ENDS-only users compared to exclusive cigarette smokers. However, some studies have found that ENDS-only users have higher concentrations of various BOE metabolites compared to non-users of tobacco products [4,6,7,9]. Comparisons of BOE metabolites in exclusive cigarette smokers compared to dual cigarette and ENDS users are not as consistent. Several studies found that dual users had similar or higher concentrations of some BOE metabolites compared to those who only used cigarettes [5,6,11], while other studies found dual users had lower levels of TSNA [8] or VOC [4] compared to exclusive cigarette smokers. Dual users have also been found to have higher levels of VOC and TSNA compared to exclusive ENDS users [5,9,11]. 

Several short-term studies of smokers or dual users measured changes in BOE metabolite levels up to 4 weeks after participants were assigned to dual use, cigarette only use, ENDS-only use, or no tobacco use, depending on the study [12,13,14,15,16,17]. All studies observed decreases in some TSNA and VOC after smokers became exclusive ENDS users [12,13,14,15,16,17]. Other studies observed no change in BOE metabolites when smokers became dual users [12,15]. However, dual users who reduced their daily cigarette consumption by at least half experienced significant decrease in VOC exposure in one study [17]. 

Understanding how changes in patterns of ENDS use affect toxicant exposure is informative for assessing the public health impact of cigarette smokers starting to use ENDS products. Using data from Wave 1 (2013–2014) and Wave 2 (2014–2015) of the Population Assessment of Tobacco and Health (PATH) Study, we assessed biomarkers of tobacco exposure among PATH Study exclusive smokers and dual users who transitioned to exclusive smoking, dual use, exclusive ENDS use, and no tobacco use at Wave 2. Given that dual users are a heterogenous group with differences in frequency of cigarette smoking (i.e., daily vs. non-daily) and changes in cigarettes smoked per day (i.e., decreasing, increasing, or no change in cigarettes per day), we also assessed changes in select BOE metabolites according to dual user cigarette smoking patterns. Our study adds to the literature by providing estimates for a broader set of biomarkers (see Supplementary Tables) after changes in the use of cigarettes and ENDS, among the U.S. population. 

## 2. Materials and Methods

### 2.1. Study Design

Data are from Wave 1 and Wave 2 Restricted Use Files (RUF) and Biomarker Restricted-Use Files (BRUF) of the PATH Study. The PATH Study is a nationally representative, longitudinal cohort study of adults and youth in the U.S., with a target population of the civilian household population of those ages 12 and older. Weights are used to produce national estimates of tobacco use and other health-related behaviors. Recruitment employed a stratified address-based, area-probability sampling design. The study was conducted by the Center for Tobacco Products (CTP), Food and Drug Administration (FDA) and the National Institute on Drug Abuse (NIDA), National Institutes of Health (NIH) under a contract with Westat [18]. Biospecimen and survey data were collected between September 2013 and December 2014 for Wave 1 and between October 2014 and October 2015 for Wave 2. All adult Wave 1 interview respondents were asked to provide urine samples, and a stratified probability sample of the 11,522 respondents who provided a sample at Wave 2 were selected for biomarker analysis. At Wave 2, 9012 of those participants provided a urine specimen that met criteria for analysis. Urine specimens were analyzed for relevant biomarkers of exposure at laboratories at the Centers for Disease Control and Prevention (CDC), National Center of Environmental Health. Laboratory results met the rigorous accuracy and precision requirements of the quality control/quality assurance program of the CDC [19]. Laboratory procedure manuals for each biomarker panel are available online (https://www.icpsr.umich.edu/icpsrweb/NAHDAP/studies/36840/datadocumentation, accessed on 22 January 2022). Additional details about the biomarker data are provided in the BRUF User Guide (http://doi.org/10.3886/ICPSR36840.userguide, accessed on 22 January 2022). Details on PATH study interview procedures, questionnaires, sampling, weighting, and data access are available at https://doi.org/10.3886/Series606 (accessed on 22 January 2022) [18]. Westat’s Institutional Review Board approved the study design and data collection protocol.

### 2.2. Analytic Sample and User Characteristics

This analysis was restricted to 2475 adults who were either exclusive cigarette smokers (*n* = 1899) or dual users of cigarettes and ENDS (*n* = 576) at Wave 1 and provided a urine sample for analysis at both Wave 1 and Wave 2. Wave 1 exclusive cigarette smokers were current cigarette smokers (i.e., smoked cigarettes every day or some days), were not current users of any other tobacco product, and had no past 3-day use of nicotine replacement therapy (NRT). Wave 1 dual users were current cigarette smokers and current ENDS users (i.e., used e-cigarettes every day or some days) who did not currently use any other tobacco products or NRT. In the Wave 2 interview, ENDS use included other electronic nicotine products in addition to e-cigarettes. Respondents were first asked if they had ever used an “electronic nicotine product,” if they responded “yes” then they were asked in separate questions if they had ever used an e-cigarette (including vape pens and personal vaporizers), e-cigar, e-hookah (including hookah pens), e-pipe, or something else (95% of ENDS users reported using e-cigarettes). At Wave 2, participants were classified into four groups: (1) exclusive cigarette smokers; (2) dual cigarette and ENDS users; (3) exclusive ENDS users; and (4) no tobacco use in the past 30-days. 

Demographic information (age, sex, race/ethnicity, and education) was collected at Wave 1. Information on frequency of tobacco product use (every day or some days) and average number of cigarettes smoked per day (CPD) was collected at Wave 1 and Wave 2. For some day smokers, CPD was based on the total reported number of cigarettes smoked per day multiplied by the number of days smoked in the past 30 days and then divided by 30. The percent change in CPD was calculated by subtracting Wave 1 CPD from Wave 2 CPD, then dividing by the Wave 1 CPD and multiplying by 100. Participants were classified as “reducers” if their CPD decreased by at least 50%, “increasers” if their CPD increased by at least 50%, and “maintainers” if their CPD changed less than 50%. CPD reductions of this magnitude have been found to be associated with reductions in some health risks [20], and these thresholds have been used with other PATH biomarker analyses of changes in CPD [21]. At Wave 2, e-cigarette users were asked whether their device was rechargeable and/or refillable. Those who used rechargeable e-cigarettes were further asked if the device used cartridges or a tank system. Devices that were rechargeable, refillable, used a tank system, and did not use cartridges were classified as “customizable.” Devices that were neither rechargeable nor refillable or used cartridges were classified as “non-customizable” devices.

### 2.3. Quantification of Biomarkers of Tobacco Exposure (BOE)

BOE encompassed several classes of chemicals including urinary metabolites of nicotine, minor tobacco alkaloids, TSNA, metals, arsenic compounds, PAH, and VOC. A complete list of the measured biomarkers and details about analytical methods and assay limits of detection (LOD) have been published previously [6]. Nine biomarkers of tobacco exposure that represent different HPHC classes were highlighted in the results because of their public health significance, consistent with a previous study [21]. Nicotine exposure was assessed using total nicotine equivalents-2 (TNE2), calculated as the molar sum of urinary cotinine and *trans*-3′-hydroxycotinine. NNAL (4-(methylnitrosamino)-1-(3-pyridyl)-1-butanol), a metabolite of NNK (4-(methylnitrosamino)-1-(3-pyridyl)-1-butanone plus its glucuronides), and NNN (N′-nitrosonornicotine plus its glucuronide) are commonly studied TSNA metabolites; NNK and NNN are classified as group I carcinogens [1]. 1-Hydroxypyrene and 2-hydroxyfluorene are PAH metabolites; 3HPMA (N-Acetyl-S- (3-hydroxypropyl)-L-cysteine), a metabolite of acrolein, 2CyEMA (N-Acetyl-S-(2-cyanoethyl)-L-cysteine), a metabolite of acrylonitrile, and 4HBeMA (N-Acetyl-S-(4-hydroxy-2-buten-1-yl)-L-cysteine), a metabolite of 1,3-butadiene were analyzed as biomarkers of VOC [22]. Lead was highlighted as a metal because it is often elevated in smokers, has been associated with several health outcomes, such as adverse cardiovascular effects, and is present in e-liquid aerosol [1,2]. Results for all 50 biomarkers are presented in Tables S1 and S2.

### 2.4. Statistical Analysis

The current analysis included 1899 Wave 1 exclusive cigarette smokers and 576 Wave 1 dual users, who also had urinary biomarker data for Wave 1 and Wave 2. Geometric mean (GM) concentrations of each biomarker were calculated and presented by Wave 2 tobacco user group. GM concentrations were creatinine corrected to control for differences in dilution of the urine samples analyzed. There were 124 individuals excluded from analyses for having creatinine values outside the range of 10–370 mg/dL, and 5 were excluded for missing creatinine data. For biomarker concentrations below the LOD, we used a value equal to the LOD divided by the square root of 2 [23]. Urinary biomarker concentrations were log-transformed to minimize the effects of data skewness on estimates. Variance estimates were assessed using balanced repeated replication with Fay’s method (Fay’s adjustment = 0.3). Statistical significance was set at *p* < 0.05. Regression models adjusted for log creatinine were used to assess whether GMs significantly changed from Wave 1 to Wave 2 for each biomarker, by Wave 2 tobacco use group. All statistical analyses were completed using SAS version 9.4 (SAS Institute Inc., Cary, NC, USA) and incorporated the appropriate sample weights to account for the complex survey design of the PATH Study. Estimates were flagged as potentially unreliable if the unweighted sample size of a non-proportion estimate or the denominator of a proportion was less than 50. An estimate was calculated from a sample where more than 40% of the biomarker values were below the LOD, or the relative standard error of an estimate was greater than 30%.

## 3. Results

Exclusive smokers who became dual users were mostly female (65.5%), non-Hispanic White (78.1%) and had a high school diploma or less as their highest education level (57.7%) (Table 1). Similar demographic characteristics were observed for dual users who became exclusive ENDS users or stopped all tobacco use, although these groups had a higher proportion of participants who had at least some college education. Wave 1 exclusive smokers who switched to exclusive ENDS use at W2 were mostly male (71.8%), non-Hispanic White (71.1%), and had some college or higher education level (53.8%).

Among Wave 1 exclusive smokers, Wave 1 CPD was higher for those who remained exclusive smokers (16.6 CPD) or transitioned to dual use (17.0 CPD) than those who became exclusive ENDS users (11.0 CPD) or stopped using all tobacco products (5.5 CPD) by Wave 2. A similar pattern was observed for Wave 1 dual users. At Wave 1, most exclusive cigarette smokers who transitioned to dual use or exclusive ENDS use were daily cigarette smokers (91.9% and 86.5%, respectively) by Wave 2. Only 35.5% of Wave 1 exclusive cigarette smokers who stopped using tobacco products at Wave 2 were daily smokers at Wave 1. Among Wave 1 dual users, 57.7% of those who became exclusive.

ENDS users, 44.0% of those who stopped using tobacco products, and 85.8% of those who went back to exclusive smoking were daily smokers at Wave 1. Most Wave 2 dual users were smoking cigarettes daily and using ENDS non-daily and only 18.8% of Wave 1 exclusive smokers who became dual users reduced their CPD 50% or more. Daily ENDS use at Wave 2 was more common in exclusive ENDS users than dual users. 

**Figure 1:**
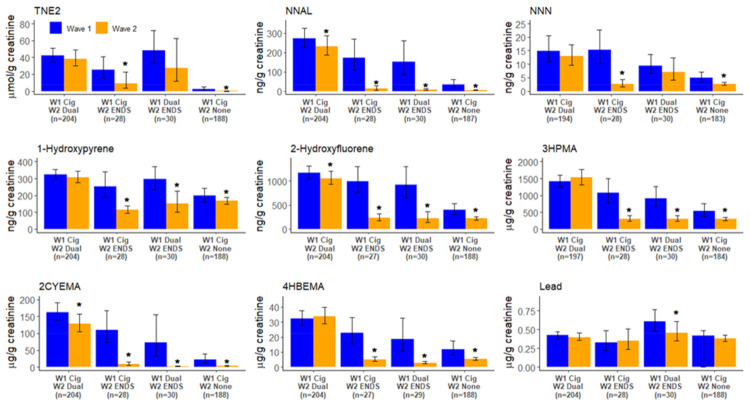
Changes of select biomarkers of exposure by tobacco use transition groups, Population Assessment of Tobacco and Health Study, Wave 1 to Wave 2 (2013–2015). Wave 1 and Wave 2 geometric means for nicotine (TNE2), 4-(methylnitrosamino)-1-(3-pyridyl)-1-butanol (NNAL), *N*’-nitrosonornicotine (NNN), 1-hydroxypyrene, 2-hydroxyfluorene, N-Acetyl-S-(3-hydroxypropyl)-L-cysteine (3HPMA) (Acrolein metabolite), N-Acetyl-S-(2-cyanoethyl)-L-cysteine (2CYEMA) (Acrylonitrile metabolite), *N*-acetyl-S-(4-hydroxy-2-buten-1-yl)-L-cysteine (4HBEMA) (1,3 butadiene), and lead by transition group. Whiskers depict 95% confidence intervals for the geometric mean. Estimates based on an unweighted sample size sample size of less than 50 or the relative standard error of the estimate is larger than 30% and should be interpreted with caution due to low statistical precision. All estimates are weighted. * Statistically significant change (*p*-value < 0.05) in the geometric mean from Wave 1 to Wave 2.

Figure 1 presents the geometric means for select biomarkers at Waves 1 and 2 in four transition categories. Among those who transitioned from exclusive smoking to dual use (*n* = 204), no significant changes were observed for concentrations of TNE2, NNN, 1-hydroxypyrene, 3HPMA, 2CyEMA, 4HBeMA, or lead. Significant decreases were observed for NNAL (15% decrease) and 2-hydroxyflourene (10% decrease), which was the only PAH that significantly decreased in this group (Table S1). Findings for the transition groups with fewer than 50 users should be interpreted with caution due to low statistical precision. Exclusive smokers who became exclusive ENDS users (*n* = 28) had significant reductions in metabolites for TNE2, all TSNA including a 93% reduction in NNAL, all PAH, and most VOC (Figure 1 and Table S1). Dual users who became exclusive ENDS users (*n* = 30) did not experience a significant change in TNE2, but did have significant reductions in most TSNA, PAH, and VOC (Figure 1 and Table S2). Exclusive smokers who transitioned to no tobacco product use (*n* = 188) had significant decreases in TNE2, all TSNA, most PAH, and most VOC (Figure 1 and Table S1). Similar reductions were observed for Wave 1 dual users who stopped using all tobacco products (*n* = 30) (Table S2). Lead levels significantly decreased among dual users who became exclusive ENDS users but did not significantly change in any other transition group. A complete list of Wave 1 and Wave 2 geometric means for all tobacco BOEs by all transitions is presented in Tables S1 and S2.

**Figure 2:**
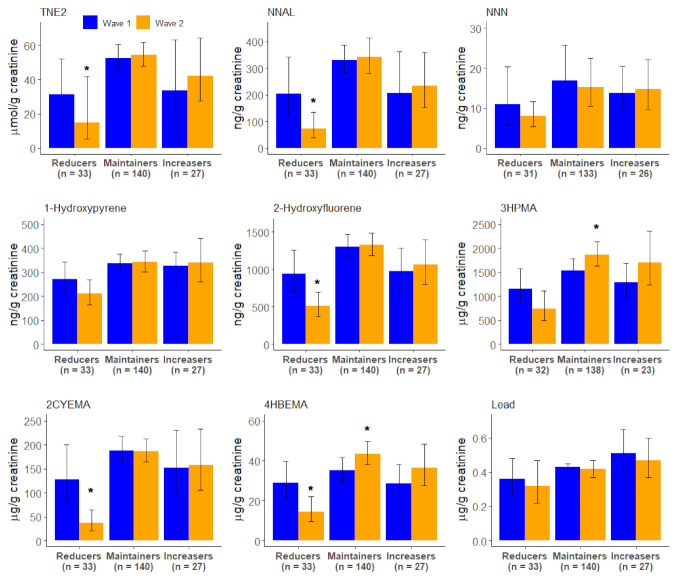
Wave 1 and Wave 2 geometric mean concentrations of select biomarkers, among exclusive cigarette smokers who became dual users (*n* = 204), by Wave 1 to Wave 2 change in cigarettes per day (CPD), Population Assessment of Tobacco and Health Study, 2013–2015. Reducers–reduced cigarettes smoked per day (CPD) by ≥50% between Wave 1 and Wave 2; Maintainers–change in CPD between Wave 1 and Wave 2 was <50%; Increasers–increased CPD by ≥50% between Wave 1 and Wave 2. GM, geometric mean; CI, confidence interval. Wave 1 and Wave 2 geometric means for nicotine (TNE2), 4-(methylnitrosamino)-1-(3-pyridyl)-1-butanol (NNAL), *N*’-nitrosonornicotine (NNN), 1-hydroxypyrene, 2-hydroxyfluorene, *N*-acetyl-S-(3-hydroxypropyl)-L-cysteine (3HPMA) (Acrolein metabolite), N-Acetyl-S-(2-cyanoethyl)-L-cysteine (2CYEMA) (Acrylonitrile metabolite), N-Acetyl-S-(4-hydroxy-2-buten-1-yl)-L-cysteine (4HBEMA) (1,3 butadiene), and lead by transition group. Whiskers depict 95% confidence intervals for the geometric mean. All estimates are weighted. * Statistically significant change (*p*-value < 0.05) in the geometric mean from Wave 1 to Wave 2.

Stratified analyses were conducted among exclusive cigarette smokers who became dual users to assess whether changes in CPD influenced changes in biomarker levels (Figure 2). Many of the dual users were either maintainers or increasers. Those who were reducers had the highest number of biomarkers which demonstrated significant reduction in concentration including TNE2, NNAL, 2-hydroxyflourene, 2CyEMA, and 4HBeMA. Those who increased their CPD by 50% or more did not display any significant changes in these biomarkers.

## 4. Discussion

Transitioning from exclusive smoking to dual use led to reductions in NNAL, but no significant decreases in nicotine metabolites or most PAHs and VOC. Furthermore, stratified analyses suggest that changes in BOEs while transitioning from exclusive smoking to dual use are influenced by changes in CPD. Specifically, significant reductions in nicotine metabolites, TSNA, PAH, and VOC, were only observed if CPD decreased by at least 50%. Therefore, smokers who also start using ENDS are more likely to experience decreases in biomarkers of tobacco-related toxicants if they reduce their cigarette smoking by at least half, which was also observed in another PATH biomarker analysis [21]. Dual users who do not reduce their CPD may not experience a reduction in many BOEs. 

In contrast, exclusive smokers who became exclusive ENDS users experienced significant reductions in nicotine metabolites as well as most TSNA, PAH, and VOC. Recent studies also found that compared to smoking, e-cigarette use only was associated with lower nicotine exposure for most participants [24,25]. However, one study did observe that nicotine exposure for using variable-power tank e-cigarette devices only was similar to smoking, while using cig-a-like or fixed-power tank devices only led to lower nicotine exposure than smoking [24]. Significant reductions in nicotine and most TSNA, PAH, and VOC were also observed in exclusive smokers who stopped using all tobacco products. Dual users who became exclusive ENDS users also experienced reductions in some TSNA and most PAH, and VOC, but did not experience significant decreases in TNE2 and NNN. Although we observed significant reductions in many VOC among smokers who switched to only using ENDS, several cross-sectional studies observed higher concentrations of several VOC in ENDS only users compared to never tobacco users [4,6,7], suggesting that exclusive ENDS users may still be exposed to more toxicants than persons who do not use tobacco. 

Fewer reductions in BOEs among smokers who become dual users compared to smokers who completely switch to ENDS is consistent with some published studies that evaluated smokers who began using ENDS [12,13,14,15]. In line with our results, several studies observed that nicotine metabolites were significantly reduced among smokers who became exclusive ENDS users, but not among smokers who became dual users [12,15,17]. Other switching studies saw no change in nicotine metabolites when smokers became exclusive ENDS users or dual users [13,14]. In line with our findings, significant reductions in NNAL were observed in all studies where smokers switched to exclusive ENDS [12,13,14,15,17] or became dual users [15,17]. Switching studies also observed reductions in most or all measured VOC in smokers who starting using ENDS exclusively [13,14,15,16,17]. Studies have also found that smokers who became dual users also experienced reductions in VOC, however, the magnitude of the reductions were often less than what was observed for complete switchers [14,15,17]. We observed significant reductions in fewer PAH than VOC for those who became exclusive ENDS users or dual users (See Supplementary Tables for full PAH results). There were significant reductions in 1-hydroxypyrene for smokers who became exclusive ENDS users, but not dual users, and reductions in 2-hydroxyfluorene for both those groups. Another switching study that saw reductions in three of eight measured PAHs when smokers started using ENDS, observed a significant decrease in levels of 2-hydroxyfluorene, but not 1-hydroxypyrene [13], while two other switching studies found decreases in 1-hydroxypyrene for both exclusive ENDS and dual users [12,15].

A primary strength of the PATH Study is that it uses weights to produce national estimates of tobacco use patterns. In addition, this analysis evaluated a broader variety of BOE than previous studies. A limitation of these data was that they were collected in 2013–2014 (Wave 1) and 2014–2015 (Wave 2). Since then, there have been changes in the types of ENDS devices available in the marketplace, with a significant increase in the use of pod system ENDS devices [26,27]. One study found that users of pod system ENDS had a significantly higher concentration of cotinine than non-pod ENDS users [28]. Therefore, smokers who switch to exclusive use of more recent ENDS devices may not experience the same reduction in nicotine exposure that we observed. Future analyses could examine changes in biomarker exposure by ENDS device type as well as CPD category. Also, the sample size of exclusive smokers and dual users who transitioned to exclusive ENDS use is small, therefore these findings need to be interpreted with caution and should be replicated. Additionally, Wave 1 to Wave 2 changes do not account for how long ago tobacco use transitions occurred (e.g., time since stopped smoking or started ENDS in the past 12 months). 

## 5. Conclusions

These findings suggest that smokers may experience reductions in exposure to several tobacco-related toxicants when they completely switch to ENDS products or become dual users and reduce the number of cigarettes smoked per day by at least half. Future analyses that incorporate biomarker data from additional PATH Study waves may provide additional information on whether levels of biomarkers of tobacco toxicants are sustained in these individuals who transitioned from exclusive cigarette smokers to dual or exclusive ENDS use. 

## Figures and Tables

**Table 1:** Characteristics of participants by Wave 2 tobacco use group, Population Assessment of Tobacco and Health Study (2013–2105) (%, 95% Confidence Interval).

	W1 Exclusive Cigarette Smokers (*n* = 1899)				W1 Dual Cigarette/ENDS Users (*n* = 576)			
Characteristic	W2 Exclusive Cigarette Use (*n* = 1479)	W2 Dual Use (*n* = 204)	W2 Exclusive ENDS Use (*n* = 28)	W2 No Tobacco Use (*n* = 188)	W2 Exclusive Cigarette Use (*n* = 273)	W2 Dual Use (*n* = 242)	W2 Exclusive ENDS Use (*n* = 30)	W2 No Tobacco Use (*n* = 31)
**Age, mean (SE)**	45.5 (0.6)	41.6 (1.4)	39.1 (3.6) ^a^	43.4 (2.1)	42.7 (0.9)	43.0 (0.9)	44.8 (3.0) ^a^	41.0 (3.4) ^a^
**Sex**								
Male	46.6 (42.5, 50.8)	34.5 (26.5, 43.5)	71.8 (53.3, 85.0) ^a^	40.6 (31.0, 51.0)	37.5 (30.6, 44.9)	37.9 (30.7, 45.6)	29.6 (13.8, 52.4) ^a^	27.9 (14.4, 47.1) ^a^
Female	53.4 (49.2, 57.5)	65.5 (56.5, 73.5)	28.2 (15.0, 46.7) ^a^	59.4 (49.0, 69.0)	62.5 (55.1, 69.4)	62.1 (54.4, 69.3)	70.4 (47.6, 86.2) ^a^	72.1 (52.9, 85.6) ^a^
**Race/ethnicity**								
Non-Hispanic White	66.0 (61.4, 70.3)	78.1 (69.5, 84.8)	71.1 (41.6, 89.4) ^a^	59.4 (47.8, 70.0)	76.7 (70.9, 81.5)	81.7 (76.3, 86.1)	86.3 (67.1, 95.1) ^a^	68.9 (48.3, 84.0) ^a^
Other ^b^	34.0 (29.7, 38.6)	21.9 (15.2, 30.5)	28.9 (10.6, 58.4) ^a^	40.6 (30.0, 52.2)	23.3 (18.5, 29.1)	18.3 (13.9, 23.7)	13.7 (4.9, 32.9) ^a^	31.1 (16.0, 51.7) ^a^
**Educational level**								
Less than HS/GED	27.3 (24.4, 30.4)	25.6 (17.8, 35.3)	18.0 (6.9, 39.3) ^a^	20.4 (14.4, 28.0)	22.5 (17.9, 27.8)	20.8 (15.8, 26.9)	23.8 (11.2, 43.5) ^a^	17.2 (6.5, 38.3) ^a^
HS graduate	31.2 (27.3, 35.4)	32.1 (22.5, 43.4)	28.3 (11.0, 55.7) ^a^	32.1 (21.1, 45.5)	26.6 (20.4, 33.9)	22.0 (17.3, 27.7)	12.0 (4.5, 28.1) ^a^	24.5 (12.2, 43.1) ^a^
Some college or higher	41.5 (37.5, 45.6)	42.3 (32.5, 52.9)	53.8 (31.5, 74.7) ^a^	47.6 (37.6, 57.7)	50.9 (44, 57.7)	57.1 (50.5, 63.5)	64.3 (42.7, 81.2) ^a^	58.3 (38.2, 76.0) ^a^
**CPD, mean (SE)**								
Wave 1	16.6 (1.1)	17.0 (2.2)	11.0 (1.8) ^a^	5.5 (0.8)	14.3 (0.7)	13.7 (0.6)	8.8 (1.8) ^a^	8.6 (2.2) ^a^
Wave 2	14.0 (0.7)	14.1 (1.0)	NA	NA	17.9 (3.0)	13.6 (0.9)	NA	NA
**W1 daily cigarette smoking**	83.7 (81.1, 86.0)	91.9 (87.3, 94.9)	86.5 (69.4, 94.7) ^a^	35.5 (24.2, 48.7)	85.8 (80.8, 89.6)	83.1 (77.8, 87.3)	57.7 (34.9, 77.7) ^a^	44.0 (26.6, 63.0) ^a^
**W2 daily cigarette smoking**	82.6 (79.0, 85.6)	82.5 (73.1, 89.1)	NA	NA	88.1 (83.3, 91.7)	76.6 (69.8, 82.2)	NA	NA
**W1-W2 change in CPD**								
Reduced CPD by ≥50%	15.0 (12.1–18.5)	18.8 (12.5–27.3)	NA	NA	8.5 (5.4–13.3)	18.7 (14.0–24.6)	NA	NA
Increased CPD by ≥50%	18.4 (15.6–21.6)	10.0 (6.6–14.9)	NA	NA	19.8 (15.4–25.1)	16.4 (12.0–21.9)	NA	NA
Change in CPD <50%	66.6 (62.5–70.4)	71.2 (62.5–78.6)	NA	NA	71.7 (65.5–77.1)	64.9 (57.9–71.3)	NA	NA
**W2 daily ENDS use**	NA	13.3 (8.2, 20.9)	80.3 (62.4, 91.0) ^a^	NA	NA	21.0 (15.6, 27.7)	85.1 (66.2, 94.4) ^a^	NA
**W2 flavored ENDS use**	NA	58.2 (48.6, 67.2)	80.5 (60.1, 91.9) ^a^	NA	NA	58.0 (49.5, 66.0)	72.8 (51.9, 86.9) ^a^	NA
**W2 ENDS device type ^c^**								
Customizable	NA	59.0 (50.1, 67.4)	70.3 (40.6, 89.1) ^a^	NA	NA	61.1 (52.1, 69.4)	74.6 (50.6, 89.4) ^a^	NA
Non-customizable	NA	41.0 (32.6, 49.9)	29.7 (10.9, 59.4) ^a^	NA	NA	38.9 (30.6, 47.9)	25.4 (10.6, 49.4) ^a^	NA
**W2 cigarette/ENDS frequency of use**								
Daily cigarette, daily ENDS	NA	7.0 (4.3, 11.4)	NA	NA	NA	10.2 (6.6, 15.4)	NA	NA
Daily cigarettes, non-daily ENDS	NA	75.4 (66.8, 82.4)	NA	NA	NA	66.4 (58.6, 73.3)	NA	NA
Non-daily cigarette, daily ENDS	NA	6.3 (2.5, 14.8)	NA	NA	NA	10.8 (7.0, 16.4)	NA	NA
Non-daily cigarette, non-daily ENDS	NA	11.2 (6.2, 19.4)	NA	NA	NA	12.6 (8.5, 18.3)	NA	NA

Abbreviations: W1, Wave 1; W2, Wave 2; HS, high school; GED, general education diploma; CPD, cigarettes per day; NA, not applicable. The estimates are weighted percentages and 95% confidence intervals. ^a^ Estimate should be interpreted with caution because it has low statistical precision. It is based on an unweighted sample size of a non-proportion estimate or the denominator of a proportion that was less than 50, or the relative standard error of the estimate is larger than 30%. ^b^ “Other” race category includes non-Hispanic Black, Hispanic, and non-Hispanic Other. Categories were combined due to small sample sizes. ^c^ Devices that were rechargeable, refillable, used a tank system, and did not use cartridges were classified as “customizable.” Devices that were neither rechargeable nor refillable or used cartridges were classified as “non-customizable” devices. Devices with other combinations of characteristics at Wave 2 were classified as “other” types of devices. Users of other electronic nicotine products other than e-cigarettes were not asked about device type.

## Data Availability

PATH Study Restricted-Use Files and Biomarker Restricted-Use Files data are available upon approved request at https://www.icpsr.umich.edu/web/NAHDAP/series/606 (accessed on 23 January 2022).

## Supplementary Materials

The following are available online at https://www.mdpi.com/article/10.3390/ijerph19031462/s1, Table S1: Geometric mean biomarker concentrations and 95% CIs by Wave 2 tobacco use status, among Wave 1 exclusive cigarette smokers, PATH Study (2013–2015), Table S2: Geometric mean biomarker concentrations and 95% CIs by Wave 2 tobacco use status, among Wave 1 dual cigarette/ENDS users, PATH Study (2013–2015).

## References

[1] Chang C.M., Edwards S.H., Arab A., Del Valle-Pinero A.Y., Yang L., Hatsukami D.K. (2017). Biomarkers of tobacco exposure: Summary of an FDA-sponsored public workshop. Cancer Epidemiol. Biomark. Prev..

[2] Goniewicz M.L., Knysak J., Gawron M., Kosmider L., Sobczak A., Kurek J., Prokopowicz A., Jablonska-Czapla M., Rosik-Dulewska C., Havel C. (2014). Levels of selected carcinogens and toxicants in vapour from electronic cigarettes. Tob. Control.

[3] De Jesús V.R., Bhandari D., Zhang L., Reese C., Capella K., Tevis D., Zhu W., Del Valle-Pinero A.Y., Lagaud G., Chang J.T. (2020). Urinary biomarkers of exposure to volatile organic compounds from the Population Assessment of Tobacco and Health Study wave 1 (2013-2014). Int. J. Environ. Res. Public Health.

[4] Keith R.J., Fetterman J.L., Orimoloye O.A., Dardari Z., Lorkiewicz P.K., Hamburg N.M., DeFilippis A.P., Blaha M.J., Bhatnagar A. (2020). Characterization of volatile organic compound metabolites in cigarette smokers, electronic nicotine device users, dual users, and nonusers of tobacco. Nicotine Tob. Res..

[5] Smith D.M., Shahab L., Blount B.C., Gawron M., Kosminder L., Sobczak A., Xia B., Sosnoff C.S., Goniewicz M.L. (2020). Differences in exposure to nicotine, tobacco-specific nitrosamines, and volatile organic compounds among electronic cigarette users, tobacco smokers, and dual users from three countries. Toxics.

[6] Goniewicz M.L., Smith D.M., Edwards K.C., Blount B.C., Caldwell K.L., Feng J., Wang L., Christensen C., Ambrose B., Borek N. (2018). Comparison of nicotine and toxicant exposure in users of electronic cigarettes and combustible cigarettes. JAMA Netw. Open.

[7] Lorkiewicz P., Riggs D.W., Keith R.J., Conklin D.J., Xie Z., Sutaria S., Lynch B., Srivastava S., Bhatnagar A. (2019). Comparison of urinary biomarkers of exposure in humans using electronic cigarettes, combustible cigarettes, and smokeless tobacco. Nicotine Tob. Res..

[8] Piper M.E., Baker T.B., Benowitz N.L., Kobinsky K.H., Jorenby D.E. (2019). Dual users compared to smokers: Demographics, dependence, and biomarkers. Nicotine Tob. Res..

[9] Rubinstein M.L., Delucchi K., Benowitz N.L., Ramo D.E. (2018). Adolescent exposure to toxic volatile organic chemicals from e-cigarettes. Pediatrics.

[10] Hecht S.S., Carmella S.G., Kotandeniya D., Pillsbury M.E., Chen M., Ransom B.W., Vogel R.I., Thompson E., Murphy S.E., Hatsukami D.K. (2015). Evaluation of toxicant and carcinogen metabolites in the urine of e-cigarette users versus cigarette smokers. Nicotine Tob. Res..

[11] Shahab L., Goniewicz M.L., Blount B.C., Brown J., McNeill A., Alwis K.U., Feng J., Wang L., West R. (2017). Nicotine, carcinogen, and toxin exposure in long-term e-cigarette and nicotine replacement therapy users: A cross-sectional study. Ann. Intern. Med..

[12] Czoli C.D., Fong G.T., Goniewicz M.L., Hammond D. (2019). Biomarkers of exposure among “dual users” of tobacco cigarettes and electronic cigarettes in Canada. Nicotine Tob. Res..

[13] Goniewicz M.L., Gawron M., Smith D.M., Peng M., Jacob P., Benowitz N.L. (2017). Exposure to nicotine and selected toxicants in cigarette smokers who switched to electronic cigarettes: A longitudinal within-subjects observational study. Nicotine Tob. Res..

[14] Pulvers K., Emami A.S., Nollen N.L., Romero D.R., Strong D.R., Benowitz N.L., Ahluwalia J.S. (2018). Tobacco consumption and toxicant exposure of cigarette smokers using electronic cigarettes. Nicotine Tob. Res..

[15] D’Ruiz C.D., Graff D.W., Robinson E. (2016). Reductions in biomarkers of exposure, impacts on smoking urge and assessment of product use and tolerability in adult smokers following partial or complete substitution of cigarettes with electronic cigarettes. BMC Public Health.

[16] McRobbie H., Phillips A., Goniewicz M.L., Smith K.M., Knight-West O., Przulj D., Hajek P. (2015). Effects of switching to electronic cigarettes with and without concurrent smoking on exposure to nicotine, carbon monoxide, and acrolein. Cancer Prev. Res..

[17] O’Connell G., Graff D.W., D’Ruiz C.D. (2016). Reductions in biomarkers of exposure (BoE) to harmful or potentially harmful constituents (HPHCs) following partial or complete substitution of cigarettes with electronic cigarettes in adult smokers. Toxicol. Mech. Methods.

[18] Hyland A., Ambrose B.K., Conway K.P., Borek N., Lambert E., Carusi C., Taylor K., Crosse S., Fong G.T., Cummings K.M. (2017). Design and methods of the Population Assessment of Tobacco and Health (PATH) Study. Tob. Control.

[19] Caudill S.P., Schleicher R.L., Pirkle J.L. (2008). Multi-rule quality control for the age-related eye disease study. Stat. Med..

[20] Chang J.T., Anic G.M., Rostron B.L., Tanwar M., Chang C.M. (2021). Cigarette Smoking Reduction and Health Risks: A Systematic Review and Meta-analysis. Nicotine Tob. Res..

[21] Rostron B.L., Corey C.G., Chang J.T., van Bemmel D.M., Miller M.E., Chang C.M. (2020). Changes in cigarettes per day and biomarkers of exposure among US adult smokers in the Population Assessment of Tobacco and Health Study waves 1 and 2 (2013-2015). Nicotine Tob. Res..

[22] International Agency for Research on Cancer (2004). Tobacco Smoke and Involuntary Smoking.

[23] Hornung R.W., Reed L.D. (1990). Estimation of average concentration in the presence of nondetectable values. Appl. Occup. Environ. Hyg..

[24] Harvanko A.M., St Helen G., Nardone N., Addo N., Benowitz N.L. (2020). Twenty-four-hour subjective and pharmacological effects of ad-libitum electronic and combustible cigarette use among dual users. Addiction.

[25] St Helen G., Nardone N., Addo N., Dempsey D., Havel C., Jacob P., Benowitz N.L. (2020). Differences in nicotine intake and effects from electronic and combustible cigarettes among dual users. Addiction.

[26] Huang J., Duan Z., Kwok J., Binns S., Vera L.E., Kim Y., Szczypka G., Emery S.L. (2019). Vaping versus JUULing: How the extraordinary growth and marketing of JUUL transformed the US retail e-cigarette market. Tob. Control.

[27] King B.A., Gammon D.G., Marynak K.L., Rogers T. (2018). Electronic cigarette sales in the United States, 2013–2017. JAMA.

[28] Boykan R., Messina C.R., Chateau G., Eliscu A., Tolentino J., Goniewicz M.L. (2019). Self-reported use of tobacco, e-cigarettes, and marijuana versus urinary biomarkers. Pediatrics.

