# Supplementary material for: Changes in Biomarkers of Tobacco Exposure among Cigarette Smokers Transitioning to ENDS Use: The Population Assessment of Tobacco and Health Study, 2013–2015
Source: Int J Environ Res Public Health. 2022 Jan 27;19(3):1462. doi: 10.3390/ijerph19031462 (PMC8835100; doi:10.3390/ijerph19031462)
Supplement: Supplementary file 1 [file ijerph-19-01462-s001.zip › ijerph-1553152-supplementary.pdf]

Table S1. Geometric mean biomarker concentrations and 95% CIs by Wave 2 tobacco use status, among Wave 1 exclusive cigarette smokers, PATH Study (2013-2015).

	Exclusive Cigarette Smoking (N = 1479)		Dual Cigarette/ENDS Use (N = 204)		Exclusive ENDS Use (N = 28)†		No Tobacco Use (N = 188)	
Biomarker	Wave 1	Wave 2	Wave 1	Wave 2	Wave 1	Wave 2	Wave 1	Wave 2
**Urinary Nicotine Metabolites (ug/g creatinine)**								
**Nicotine Equivalence (TNE2) (μmol/g creatinine)**								
Mean	31.5	31.2	42.3	38.5	25.1	9.1*	2.3†	0.1*
(95% CI)	(28.0, 35.5)	(28.0, 34.8)	(35.1, 50.9)	(30.3, 48.9)	(15.3, 41.0)	(3.6, 22.9)	(1.0, 5.4)	(0.0, 0.1)
p-value		0.34		0.31		0.0093		<.0001
% change		-1.1%		-8.9%		-63.9%		-97.0%
**trans-3'-Hydroxycotinine (HCTT)**								
Mean	3528.7	3483.5	4746.8	4365.7	2503.9	933.1*	258.7	8.2*
(95% CI)	(3091.1, 4028.3)	(3074.2, 3947.2)	(3942.0, 5716.0)	(3384.0, 5632.2)	(1453.9, 4312)	(346.9, 2510.2)	(110.6, 605.3)	(4.8, 14.2)
p-value		0.39		0.41		0.011		<.0001
% change		-1.3%		-8.0%		-62.7%		-96.8%
**Cotinine (COTT)**								
Mean	2008.2	1970.3	2856.1	2510.0	1752.6	579.7*	153.6	4.5*
(95% CI)	(1792.9, 2249.2)	(1778.0, 2183.4)	(2341.3, 3484.1)	(1980.0, 3181.9)	(984.4, 3120.2)	(223.3, 1505)	(64.1, 367.8)	(2.6, 7.7)
p-value		0.17		0.13		0.0063		<.0001
% change		-1.9%		-12.1%		-66.9%		-97.1%
**Nicotine (NICT)**								
Mean	1170.8	1239.7	1381.2	1535.9	1053.4	294.1*	619.8	88.3*
(95% CI)	(1020.1, 1343.8)	(1093.0, 1405.9)	(1067.1, 1787.8)	(1260.1, 1871.9)	(542.4, 2046.0)	(140.0, 618.1)	(256.4, 1498.2)	(30.5, 255.9)
p-value		0.37		0.30		0.0024		0.0045
% change		5.9%		11.2%		-72.1%		-85.7%
**Cotinine N-oxide (COXT)**								
Mean	320.4	329.5	387.1	384.3	278.8	144.7	206.9	25.7*
(95% CI)	(295.4, 347.5)	(309.4, 350.9)	(339.1, 441.9)	(328.6, 449.4)	(197.9, 392.9)	(70.6, 296.6)	(112.3, 381.2)	(7.4, 88.5)
p-value		0.45		0.97		0.091		0.0041
% change		2.8%		-0.7%		-48.1%		-87.6%
**Nicotine 1'-oxide (NOXT)**								
Mean	352.1	357.7	435.6	461.7	402.7	142.0	185.3	20.5
(95% CI)	(318.5, 389.3)	(324.1, 394.8)	(353, 537.4)	(388.6, 548.6)	(237.4, 682.9)	(73.1, 275.9)	(75.4, 455.2)	(4.9, 85.5)
p-value		0.90		0.33		0.0032		0.016
% change		1.6%		6.0%		-64.7%		-88.9%
**Nornicotine (NNCT)**								

Biomarker	Exclusive Cigarette Smoking (N = 1479) Wave 1	Exclusive Cigarette Smoking (N = 1479) Wave 2	Dual Cigarette/ENDS Use (N = 204) Wave 1	Dual Cigarette/ENDS Use (N = 204) Wave 2	Exclusive ENDS Use (N = 28)† Wave 1	Exclusive ENDS Use (N = 28)† Wave 2	No Tobacco Use (N = 188) Wave 1	No Tobacco Use (N = 188) Wave 2
Mean	64.3	66.8	73.9	73.5	60.1	18.9*	36.9	7.4*
(95% CI)	(58.2, 71.1)	(60.9, 73.4)	(61.7, 88.5)	(63.7, 84.8)	(40.5, 89.2)	(10.6, 34)	(18.6, 72.9)	(3.0, 18.1)
p-value		0.38		0.91		0.0024		0.0052
% change		3.9%		-0.6%		-68.5%		-80.0%
**Minor Tobacco Alkaloids (ug/g creatinine)**								
**Anabasine (ANBT)**								
Mean	8.5	8.4	9.4	8.3	7.6	0.8*†	4.6	1.2*†
(95% CI)	(7.6, 9.4)	(7.6, 9.2)	(7.7, 11.5)	(6.9, 10.0)	(4.7, 12.3)	(0.4, 1.7)	(2.7, 8.1)	(0.6, 2.6)
p-value		0.29		0.27		<.0001		0.0039
% change		-1.2%		-11.9%		-88.9%		-74.1%
**Anatabine (ANTT)**								
Mean	14.1	14.5	16.1	13.9	12.4	0.7*†	7.0	1.3*†
(95% CI)	(12.5, 15.9)	(13.0, 16.3)	(12.9, 20.2)	(11.0, 17.7)	(7.1, 21.7)	(0.4, 1.6)	(3.7, 13.2)	(0.5, 3.3)
p-value		0.56		0.31		<.0001		0.0029
% change		3.5%		-13.7%		-94.0%		-81.9%
**Tobacco Specific Nitrosamines (TSNAs) (ng/g creatinine)**								
**4-methylnitrosamino)-4-(3-pyridyl)-1-butanol (NNAL)**								
Mean	216.9	218.1	272.0	231.9*	172.5	12.5*	33.5	5.0*
(95% CI)	(195.9, 240.2)	(199.2, 238.8)	(226.5, 326.6)	(187.0, 287.5)	(110.2, 269.8)	(5.7, 27.3)	(18.7, 60.2)	(3.6, 7.0)
p-value		0.52		0.037		<.0001		<.0001
% change		0.5%		-14.8%		-92.8%		-85.0%
**N'-Nitrosonornicotine (NNN)**								
Mean	11.9	11.6	14.9	12.9	15.3	2.6*	4.9	2.7*
(95% CI)	(10.9, 12.9)	(10.4, 13)	(10.8, 20.5)	(9.7, 17.2)	(10.3, 22.7)	(1.5, 4.4)	(3.4, 7.0)	(2.3, 3.2)
p-value		0.23		0.15		<.0001		<.0001
% change		-1.9%		-13.4%		-83.3%		-44.4%
**N’-nitrosoanatabine (NAT)**								
Mean	97.9	104.9	132.0	115.4	55.5	3.1*	19.8	4.9*
(95% CI)	(86.1, 111.4)	(93.4, 117.7)	(101.5, 171.7)	(89.7, 148.6)	(18.5, 166.1)	(1.9, 5.1)	(12, 32.8)	(3.9, 6.2)
p-value		0.23		0.29		<.0001		<.0001
% change		7.1%		-12.6%		-94.4%		-75.2%
**N’-nitrosoanabasine (NAB)**								
Mean	16.0	15.8	21.0	17.2*	10.3	1.2*†	4.5	1.6*

Biomarker	Exclusive Cigarette Smoking (N = 1479) Wave 1	Wave 2	Dual Cigarette/ENDS Use (N = 204) Wave 1	Wave 2	Exclusive ENDS Use (N = 28)† Wave 1	Wave 2	No Tobacco Use (N = 188) Wave 1	Wave 2
(95% CI)	(14.1, 18.1)	(14.1, 17.6)	(16.5, 26.6)	(14.0, 21.1)	(4.1, 25.7)	(0.7, 1.9)	(3.1, 6.8)	(1.3, 1.9)
p-value		0.45		0.041		<.0001		<.0001
% change		-1.1%		-18.0%		-88.7%		-65.4%
**Heavy Metals (ug/g creatinine)**								
**Beryllium**								
Mean	0.01	0.01*	0.01	0.01*	0.01	0.01	0.01	0.01*
(95% CI)	(0.01, 0.01)	(0.01, 0.01)	(0.01, 0.01)	(0.01, 0.01)	(0.01, 0.01)	(0.01, 0.01)	(0.01, 0.02)	(0.01, 0.01)
p-value		0.0006		0.041		0.71		0.043
% change		0.0%		0.0%		0.0%		0.0%
**Cadmium**								
Mean	0.30	0.32	0.29	0.29	0.20	0.20	0.19	0.19
(95% CI)	(0.28, 0.33)	(0.29, 0.34)	(0.25, 0.35)	(0.23, 0.35)	(0.13, 0.30)	(0.14, 0.30)	(0.16, 0.23)	(0.16, 0.23)
p-value		0.17		0.82		0.46		0.66
% change		6.7%		0.0%		0.0%		0.0%
**Cobalt**								
Mean	0.53	0.53	0.53	0.51	0.45	0.45	0.54	0.64†
(95% CI)	(0.51, 0.55)	(0.51, 0.56)	(0.47, 0.59)	(0.46, 0.57)	(0.34, 0.6)	(0.37, 0.56)	(0.47, 0.62)	(0.48, 0.86)
p-value		0.93		0.73		0.61		0.22
% change		0.0%		-3.8%		0.0%		18.5%
**Lead**								
Mean	0.49	0.49	0.43	0.40	0.33	0.35	0.42	0.38
(95% CI)	(0.47, 0.51)	(0.47, 0.52)	(0.38, 0.47)	(0.36, 0.46)	(0.22, 0.49)	(0.24, 0.51)	(0.37, 0.49)	(0.34, 0.43)
p-value		0.77		0.22		0.12		0.056
% change		0.0%		-7.0%		6.1%		-9.5%
**Manganese**								
Mean	0.14	0.16*	0.13	0.14	0.11	0.09	0.13	0.16*
(95% CI)	(0.13, 0.14)	(0.15, 0.18)	(0.12, 0.15)	(0.12, 0.17)	(0.07, 0.18)	(0.07, 0.13)	(0.11, 0.16)	(0.14, 0.19)
p-value		<.0001		0.28		0.77		0.039
% change		14.3%		7.7%		-18.2%		23.1%
**Strontium**								
Mean	108.38	111.94	125.10	124.30	90.62	98.07	111.10	119.51
(95% CI)	(99.81, 117.67)	(104.96, 119.37)	(110.33, 141.85)	(110.58, 139.74)	(69.54, 118.09)	(78.95, 121.82)	(96.58, 127.79)	(105.55, 135.32)
p-value		0.66		0.97		0.40		0.31
% change		3.3%		-0.6%		8.2%		7.6%

Biomarker	Exclusive Cigarette Smoking (N = 1479) Wave 1	Exclusive Cigarette Smoking (N = 1479) Wave 2	Dual Cigarette/ENDS Use (N = 204) Wave 1	Dual Cigarette/ENDS Use (N = 204) Wave 2	Exclusive ENDS Use (N = 28)† Wave 1	Exclusive ENDS Use (N = 28)† Wave 2	No Tobacco Use (N = 188) Wave 1	No Tobacco Use (N = 188) Wave 2
**Thallium**								
Mean	0.15	0.16	0.15	0.15	0.15	0.17*	0.17	0.18
(95% CI)	(0.14, 0.16)	(0.15, 0.17)	(0.14, 0.17)	(0.14, 0.17)	(0.13, 0.17)	(0.13, 0.22)	(0.15, 0.18)	(0.16, 0.20)
p-value		0.27		0.93		0.044		0.41
% change		6.7%		0.0%		13.3%		5.9%
**Uranium**								
Mean	0.01	0.01	0.01	0.01	0.00	0.00	0.01	0.01
(95% CI)	(0.01, 0.01)	(0.01, 0.01)	(0.01, 0.01)	(0.01, 0.01)	(0.00, 0.01)	(0.00, 0.01)	(0.01, 0.01)	(0.00, 0.01)
p-value		0.073		0.89		0.64		0.14
% change		0.0%		0.0%		0.0%		0.0%
**Arsenic and Arsenic Compounds (ug/g creatinine)**								
**Total Inorganic Arsenic**								
Mean	4.7	4.3	4.5	3.8	3.8	3.1*	5.4	4.8*
(95% CI)	(4.5, 4.9)	(4.1, 4.5)	(4.1, 4.9)	(3.4, 4.3)	(2.8, 5.1)	(2.4, 4.1)	(4.8, 6.0)	(4.2, 5.4)
p-value		<.0001		0.0004		0.19		0.0060
% change		-8.5%		-15.6%		-18.4%		-11.11%
**Polycyclic Aromatic Hydrocarbons (ng/g creatinine)§**								
**1-Naphthol or 1-hydroxynaphthalene (1-NAP) (ug/g)**								
Mean	11.4	12.0	14.1	13.4	9.5	2.9*†	3.6	2.0*
(95% CI)	(10.4, 12.6)	(11.0, 13.0)	(9.7, 20.4)	(9.0, 19.9)	(5.2, 17.5)	(1.3, 6.4)	(2.5, 5.3)	(1.5, 2.8)
p-value		0.35		0.66		0.03		0.0019
% change		4.7%		-4.6%		-69.8%		-43.6%
**2-Naphthol or 2-hydroxynaphthalene (2-NAP) (ug/g)**								
Mean	14.4	14.9	15.6	14.4	11.3	4.2*	7.8	6.3*
(95% CI)	(13.6, 15.1)	(14.1, 15.7)	(13.9, 17.6)	(12.8, 16.1)	(8.3, 15.5)	(3.4, 5.1)	(6.4, 9.6)	(5.4, 7.3)
p-value		0.12		0.066		<.0001		0.0069
% change		3.7%		-8.1%		-62.9%		-19.6%
**3-Hydroxyfluorene (3-FLU)**								
Mean	595.3	624.3	679.1	619.5	537.6	122.7*	194.1	91.5*
(95% CI)	(555.6, 637.8)	(587.9, 662.9)	(603.1, 764.7)	(539.9, 710.8)	(398.5, 725.4)	(80.4, 187.2)	(141.0, 267.3)	(74.2, 112.7)
p-value		0.07		0.11		<.0001		<.0001
% change		4.9%		-8.8%		-77.2%		-52.9%
**2-Hydroxyfluorene (2-FLU)**								

Biomarker	Exclusive Cigarette Smoking (N = 1479) Wave 1	Exclusive Cigarette Smoking (N = 1479) Wave 2	Dual Cigarette/ENDS Use (N = 204) Wave 1	Dual Cigarette/ENDS Use (N = 204) Wave 2	Exclusive ENDS Use (N = 28)† Wave 1	Exclusive ENDS Use (N = 28)† Wave 2	No Tobacco Use (N = 188) Wave 1	No Tobacco Use (N = 188) Wave 2
Mean	1049.0	1072.3	1172.4	1055.6*	996.7	240.2*	401.5	222.4*
(95% CI)	(984.2, 1118.0)	(1011.6, 1136.6)	(1049.9, 1309.2)	(926.6, 1202.5)	(764.9, 1298.8)	(180.8, 319.1)	(303.7, 530.8)	(188.4, 262.6)
p-value		0.60		0.044		<.0001		<.0001
% change		2.2%		-10.0%		-75.9%		-44.6%
**1-Hydroxyphenanthrene (1-PHE)**								
Mean	179.9	198.1*	190.0	195.5	141.3	93.3*	122.6	123.7
(95% CI)	(170.2, 190.3)	(188.3, 208.6)	(171.3, 210.7)	(177.7, 215.2)	(104.1, 191.8)	(79.0, 110.1)	(106.3, 141.3)	(104.6, 146.2)
p-value		<.0001		0.36		0.040		1.00
% change		10.1%		2.9%		-34.0%		0.9%
**1-Hydroxypyrene (1-PYR)**								
Mean	306.0	316.8	323.2	308.1	253.4	113.4*	197.9	167.9*
(95% CI)	(287.4, 325.9)	(298.3, 336.4)	(295.1, 354.0)	(277.4, 342.2)	(188.0, 341.5)	(93.0, 138.4)	(161.8, 242.1)	(148.9, 189.4)
p-value		0.36		0.20		<.0001		0.0074
% change		3.5%		-4.7%		-55.2%		-15.2%
**Sum of 2-Hydroxyphenanthrene and 3-Hydroxyphenanthrene (2-3PHE)**								
Mean	306.1	310.4	326.8	304.2	271.4	132.2*	177.6	144.3*
(95% CI)	(288.6, 324.8)	(294.1, 327.6)	(298.5, 357.8)	(277.1, 334.0)	(213.5, 345.1)	(110.2, 158.5)	(147.7, 213.5)	(127.3, 163.7)
p-value		0.88		0.11		<.0001		0.0078
% change		1.4%		-6.9%		-51.3%		-18.7%
**Volatile Organic Compound Metabolites (VOCM) (ug/g creatinine)**								
**2-Methylhippuric acid (2MHA) (Xylene)**								
Mean	102.7	123.9	115.6	112.6	95.9	43.5	43.2	27.6
(95% CI)	(95.3, 110.7)	(116.2, 132.0)	(98.9, 135.0)	(97.4, 130.1)	(60.9, 151.1)	(29.6, 64)	(32.3, 57.8)	(23.6, 32.3)
p-value		<.0001		0.85		0.0240		<.0001
% change		20.6%		-2.6%		-54.7%		-36.2%
**3- & 4-Methylhippuric acid (3MHA/4MHA) (Xylene)**								
Mean	687.9	707.0	755.6	704.3	589.6	226.2*	306.3	182.2*
(95% CI)	(636.1, 743.9)	(657.1, 760.7)	(662.8, 861.4)	(612.3, 810.2)	(338.3, 1027.8)	(156.2, 327.7)	(238.3, 393.8)	(145.5, 228.1)
p-value		0.54		0.26		0.010		<.0001
% change		2.8%		-6.8%		-61.6%		-40.5%
**N-Acetyl-S-(2-carbamoylethyl)-L-cysteine (2CaEMA) (Acrylamide)**								
Mean	137.9	136.5*	142.5	133.6	111.5	57.5*	88.0	59.1*
(95% CI)	(131.2, 144.9)	(130.1, 143.3)	(126.9, 160.0)	(120.2, 148.6)	(81.1, 153.3)	(48.5, 68.3)	(76.2, 101.7)	(52.4, 66.6)
p-value		0.45		0.31		0.0004		<.0001

Biomarker	Exclusive Cigarette Smoking (N = 1479) Wave 1	Exclusive Cigarette Smoking (N = 1479) Wave 2	Dual Cigarette/ENDS Use (N = 204) Wave 1	Dual Cigarette/ENDS Use (N = 204) Wave 2	Exclusive ENDS Use (N = 28)† Wave 1	Exclusive ENDS Use (N = 28)† Wave 2	No Tobacco Use (N = 188) Wave 1	No Tobacco Use (N = 188) Wave 2
% change		-1.0%		-6.2%		-48.4%		-32.9%
**N-Acetyl-S-(Nmethylcarbamoyl)-L-cysteine (MCaMA) (N,N-Dimethylformamide/isocyanates)**								
Mean	494.4	506.4	525.9	538.4*	326.4	157.2*	250.6	164.3*
(95% CI)	(463.9, 526.8)	(479.6, 534.8)	(451.1, 613.2)	(472.5, 613.4)	(218.3, 488.2)	(117.2, 210.8)	(188.8, 332.8)	(137.1, 197)
p-value		0.75		0.55		<.0001		<.0001
% change		2.4%		2.4%		-51.8%		-34.4%
**N-Acetyl-S-(benzyl)-L-cysteine (BzMA) (Toluene)**								
Mean	6.6	6.8	6.2	6.3	6.3	7.0	6.4	5.8*
(95% CI)	(6.2, 7.0)	(6.3, 7.5)	(5.6, 6.9)	(5.5, 7.3)	(4.7, 8.5)	(4.8, 10.1)	(5.6, 7.3)	(5.2, 6.4)
p-value		0.54		0.78		0.44		0.21
% change		4.1%		1.4%		10.8%		-9.6%
**N-Acetyl-S-(2-carboxyethyl)-L-cysteine (2CoEMA) (Acrolein)**								
Mean	284.2	284.2	301.1	314.9	250.0	110.4*	155.8	98.9*
(95% CI)	(262.0, 308.3)	(264.2, 305.6)	(255.7, 354.7)	(271.7, 364.8)	(183.3, 341.0)	(78.7, 154.9)	(124.7, 194.6)	(88.2, 110.9)
p-value		0.76		0.51		0.0031		0.0001
% change		0.0%		4.6%		-55.8%		-36.5%
**N-Acetyl-S-(1-cyano-2-hydroxyethyl)-L-cysteine (1CyHEMA) (Acrylonitrile)**								
Mean	22.3	24.0	27.3	25.6	19.1	2.5*	6.4	2.9*
(95% CI)	(20.2, 24.7)	(21.8, 26.4)	(21.1, 35.2)	(19.9, 33)	(12.1, 30.3)	(1.9, 3.4)	(4.3, 9.7)	(2.4, 3.5)
p-value		0.04		0.67		<.0001		<.0001
% change		7.3%		-6.1%		-86.9%		-54.5%
**N-Acetyl-S-(2-cyanoethyl)-L-cysteine (2CyEMA) (Acrylonitrile)**								
Mean	130.4	131.7	163.1	128.1*	110.2	8.6*	22.9	3.9*
(95% CI)	(118.6, 143.3)	(121.1, 143.2)	(139.4, 190.8)	(104.7, 156.6)	(72.8, 166.8)	(4.9, 14.9)	(13.3, 39.4)	(2.9, 5.3)
p-value		0.77		0.0071		<.0001		<.0001
% change		1.0%		-21.5%		-92.2%		-82.9%
**N-Acetyl-S-(3,4-dihydroxybutyl)-L-cysteine (34HBMA) (1,3 Butadiene)**								
Mean	508.2	508.6*	490.1	520.5	413.8	376.7*	433.4	358.2*
(95% CI)	(487.9, 529.5)	(492.0, 525.8)	(446.3, 538.1)	(474.8, 570.6)	(336.6, 508.6)	(296.4, 478.8)	(378.0, 496.9)	(326.0, 393.6)
p-value		0.67		0.15		0.73		0.0070
% change		0.1%		6.2%		-9.0%		-17.4%
**N-Acetyl-S-(2-carbamoyl-2-hydroxyethyl)-L-cysteine (2CaHEMA) (Acrylamide)**								
Mean	17.5	18.0	17.7	18.3	14.0	8.2*	12.1	10.2*
(95% CI)	(16.7, 18.3)	(17.1, 19.0)	(15.6, 20.1)	(16.2, 20.6)	(10.0, 19.6)	(6.6, 10.2)	(10.3, 14.3)	(9.0, 11.6)
p-value		0.58		0.68		0.0011		0.0011

	Exclusive Cigarette Smoking (N = 1479)		Dual Cigarette/ENDS Use (N = 204)		Exclusive ENDS Use (N = 28)†		No Tobacco Use (N = 188)	
Biomarker	Wave 1	Wave 2	Wave 1	Wave 2	Wave 1	Wave 2	Wave 1	Wave 2
% change		3.1%		3.0%		-41.3%		-15.6%
**N-Acetyl-S-(2-hydroxyethyl)-L-cysteine (2HEMA) (Acrylonitrile, vinyl chloride, ethylene oxide)**								
Mean	2.8	2.9	3.6	3.2	2.5	0.9*†	1.7†	0.9*†
(95% CI)	(2.6, 3.1)	(2.7, 3.2)	(2.8, 4.6)	(2.5, 4.1)	(2.0, 3.1)	(0.7, 1.1)	(1.3, 2.4)	(0.8, 1.1)
p-value		0.57		0.31		<.0001		<.0001
% change		4.3%		-10.8%		-65.7%		-45.7%
**N-Acetyl-S-(2-hydroxypropyl)-L-cysteine (2HPMA) (Propylene Oxide)**								
Mean	72.0	74.0*	79.9	83.5	60.8	38.3	44.9	31.6*
(95% CI)	(67.4, 77.0)	(69.5, 78.7)	(69.5, 91.9)	(70.6, 98.9)	(46.3, 79.9)	(23.5, 62.3)	(37.0, 54.6)	(26.5, 37.7)
p-value		0.62		0.45		0.17		0.0082
% change		2.7%		4.5%		-37.1%		-29.6%
**N-Acetyl-S-(3-hydroxypropyl)-L-cysteine (3HPMA) (Acrolein)**								
Mean	1162.5	1342.2*	1410.3	1531.6	1074.8	303.8*	544.2	299.9*
(95% CI)	(1061.0, 1273.8)	(1247.8, 1443.7)	(1240.3, 1603.6)	(1321.6, 1774.9)	(770.3, 1499.5)	(228.7, 403.7)	(390.9, 757.7)	(255.8, 351.5)
p-value		<.0001		0.26		<.0001		0.0010
% change		15.5%		8.6%		-71.7%		-44.9%
**N-Acetyl-S-(3-hydroxypropyl-1-methyl)-L-cysteine (3HMPMA) (Crotonaldehyde)**								
Mean	1353.1	1418.6	1580.6	1530.1	1186.2	245.3	559.8	260.4
(95% CI)	(1231.8, 1486.3)	(1315.9, 1529.3)	(1357.8, 184.0)	(1340.2, 1746.9)	(771.2, 1824.4)	(188.0, 320.1)	(408.7, 766.8)	(228.0, 297.4)
p-value		0.31		0.69		<.0001		<.0001
% change		4.8%		-3.2%		-79.3%		-53.5%
**N-Acetyl-S-(4-hydroxy-2-methyl-2-buten-1-yl)-L-cysteine (4HMBeMA) (Isoprene)**								
Mean	34.9	37.8*	42.4	39.1*	28.0	4.7	10.5	5.8
(95% CI)	(31.7, 38.5)	(34.3, 41.5)	(35.8, 50.3)	(31.1, 49.2)	(18.0, 43.5)	(2.9, 7.6)	(6.2, 18.0)	(4.8, 6.9)
p-value		0.07		0.53		<.0001		0.0027
% change		8.1%		-7.8%		-83.3%		-45.1%
**Mandelic acid (MADA)**								
Mean	284.4	306.3*	305.1	293.7	214.4	132.1	178.0	160.4*
(95% CI)	(271.6, 297.9)	(294.1, 319.0)	(272.8, 341.2)	(256.5, 336.3)	(123.9, 371.0)	(103.8, 168.2)	(156.0, 203.1)	(148.7, 172.9)
p-value		0.03		0.66		0.08		0.04
% change		7.7%		-3.7%		-38.4%		-9.9%
**N-Acetyl-S-(4-hydroxy-2-buten-1-yl)-L-cysteine (4HBeMA) (1,3 Butadiene)**								
Mean	28.5	31.8*	32.3	33.9	22.8	5.1*	11.8	5.4*
(95% CI)	(26.2, 31.0)	(29.6, 34.2)	(27.8, 37.6)	(28.9, 39.8)	(15.7, 33.1)	(3.9, 6.8)	(7.9, 17.5)	(4.6, 6.4)
p-value		0.0011		0.52		<.0001		0.0001

	Exclusive Cigarette Smoking (N = 1479)		Dual Cigarette/ENDS Use (N = 204)		Exclusive ENDS Use (N = 28)†		No Tobacco Use (N = 188)	
Biomarker	Wave 1	Wave 2	Wave 1	Wave 2	Wave 1	Wave 2	Wave 1	Wave 2
% change		11.7%		4.8%		-77.4%		-53.7%
**Phenylglyoxylic acid (PhGA) (Ethylbenzene, styrene)**								
Mean	380.9	404.5*	373.9	398.5	331.0	216.7*	258.3	224.2*
(95% CI)	(363.8, 398.9)	(387.4, 422.4)	(344.8, 405.5)	(360.3, 440.7)	(232.2, 471.9)	(173.4, 270.8)	(221.8, 300.9)	(197.6, 254.3)
p-value		0.03		0.12		0.02		0.03
% change		6.2%		6.6%		-34.5%		-13.2%
**N-Acetyl-S-(phenyl)-L-cysteine (PhMA) (Benzene)**								
Mean	1.1†	1.0*†	1.1†	0.9*†	1.0†	0.8†	1.0†	0.9†
(95% CI)	(1.0, 1.2)	(0.9, 1.0)	(1.0, 1.3)	(0.8, 1.0)	(0.7, 1.3)	(0.6, 1.0)	(0.8, 1.1)	(0.8, 1.0)
p-value		0.0002		0.0059		0.28		0.31
% change		-10.9%		-18.2%		-21.9%		-7.3%
**2-Thioxothiazolidine-4-carboxylic acid (TTCA) (Carbon Disulfide)**								
Mean	21.3	17.0*	22.2	19.5	18.5	18.0	22.1	14.4*
(95% CI)	(19.7, 22.9)	(15.5, 18.7)	(18.3, 26.8)	(15.1, 25.1)	(11.5, 29.8)	(10.1, 32.0)	(16.2, 30.1)	(11.3, 18.4)
p-value		<.0001		0.44		0.91		0.0025
% change		-20.0%		-12.2%		-2.8%		-34.7%

Abbreviations: : PATH – Population Assessment of Tobacco and Health, ENDS – electronic nicotine delivery system, CI – confidence interval. All estimates are weighted.

All geometric means are creatinine adjusted. P-values are for the Wave 1 to Wave 2 change in biomarker levels. % change indicates the Wave 1 to Wave 2 change in geometric mean by Wave 2 tobacco product use group.

* Indicates a statistically significant change ($p < 0.05$) in biomarker levels from Wave 1 to Wave 2.

† Estimate should be interpreted with caution because it has low statistical precision. The coefficient of variation of the estimate is larger than 30% or it is based on a denominator sample size of less than 50.

§Concentrations of 1-NAP and 2-NAP are ug/g, all other polycyclic aromatic hydrocarbon biomarker concentrations are in ng/g.

Table S2. Geometric mean biomarker concentrations and 95% CIs by Wave 2 tobacco use status, among Wave 1 dual cigarette/ENDS users, PATH Study (2013-2015).

	Exclusive Cigarette Smoking (N = 273)		Dual Cigarette/ENDS Use (N = 242)		Exclusive ENDS Use (N = 30)†		No Tobacco Use (N = 31)†	
Biomarker	Wave 1	Wave 2	Wave 1	Wave 2	Wave 1	Wave 2	Wave 1	Wave 2
**Urinary Nicotine Metabolites (ug/g creatinine)**								
**Nicotine Equivalence (TNE2) (umol/g)**								
Mean	43.3	42.1	41.5	44.8	48.4	27.6	2.9†	0.0*
(95% CI)	(36.9, 50.9)	(35.7, 49.7)	(35.2, 49.1)	(39.3, 51.2)	(32.7, 71.7)	(12.1, 62.6)	(0.7, 12.6)	(0.0, 0.1)
p-value		0.92		0.27		0.079		<.0001
% change		-2.7%		7.9%		-43.1%		-98.6%
**trans-3'-Hydroxycotinine (HCTT)**								
Mean	4783.2	4744.7	4550.2	4875.5	4854.5	2808.9	346.2	4.8*
(95% CI)	(4038.4, 5665.5)	(4020.1, 5599.8)	(3808.9, 5435.8)	(4241.6, 5604.1)	(3202, 7359.8)	(1197.6, 6588.3)	(79.9, 1499.5)	(1.9, 12.1)
p-value		0.78		0.36		0.095		<.0001
% change		-0.8%		7.1%		-42.1%		-98.6%
**Cotinine (COTT)**								
Mean	2788.8	2683.9	2786.0	3030.9	3718.1	2072.4	171.4	2.1*†
(95% CI)	(2306.4, 3372.0)	(2218.9, 3246.3)	(2352.9, 3299.0)	(2639.2, 3480.7)	(2456.0, 5628.6)	(910.4, 4717.6)	(38.4, 765.4)	(0.8, 5.3)
p-value		0.96		0.22		0.067		<.0001
% change		-3.8%		8.8%		-44.3%		-98.8%
**Nicotine (NICT)**								
Mean	1488.1	1194.1*	1296.5	1437.8	1557.0	1415.4	150.0	46.7
(95% CI)	(1175.4, 1884.0)	(955.9, 1491.6)	(1047.4, 1604.9)	(1175.1, 1759.3)	(1121.7, 2161.2)	(724.7, 2764.4)	(18.8, 1197.6)	(6.6, 329.3)
p-value		0.038		0.19		0.60		0.23
% change		-19.8%		10.9%		-9.1%		-68.9%
**Cotinine N-oxide (COXT)**								
Mean	344.2	346.6	355.2	379.3	458.5	310.5	97.0	31.6
(95% CI)	(307.1, 385.8)	(309.3, 388.3)	(316.0, 399.3)	(333.5, 431.4)	(367.3, 572.3)	(166.1, 580.4)	(20.4, 461.5)	(4.9, 205.1)
p-value		0.49		0.15		0.13		0.21
% change		0.7%		6.8%		-32.3%		-67.4%
**Nicotine 1'-oxide (NOXT)**								
Mean	399.6	336.8*	390.0	399.2	607.5	433.3	59.2	18.1†
(95% CI)	(337.2, 473.7)	(287.9, 393.9)	(326.7, 465.5)	(333.1, 478.4)	(450.3, 819.6)	(209.7, 895.5)	(5.3, 665.3)	(2.2, 152.5)
p-value		0.034		0.64		0.23		0.33
% change		-15.7%		2.4%		-28.7%		-69.4%
**Nornicotine (NNCT)**								

	Exclusive Cigarette Smoking (N = 273)		Dual Cigarette/ENDS Use (N = 242)		Exclusive ENDS Use (N = 30)†		No Tobacco Use (N = 31)†	
Biomarker	Wave 1	Wave 2	Wave 1	Wave 2	Wave 1	Wave 2	Wave 1	Wave 2
Mean	71.2	66.7	69.0	73.3	69.8	47.5	14.5	6.5†
(95% CI)	(60.5, 83.8)	(56.9, 78.1)	(60.0, 79.4)	(64.4, 83.5)	(57.1, 85.3)	(30.1, 74.8)	(3.5, 61.0)	(1.2, 35.2)
p-value		0.60		0.19		0.094		0.21
% change		-6.4%		6.2%		-32.0%		-55.0%
				**Minor Tobacco Alkaloids (ug/g creatinine)**				
**Anabasine (ANBT)**								
Mean	9.5	8.4	8.3	8.6	4.8	1.4*†	2.1†	1.1†
(95% CI)	(8.0, 11.2)	(7.1, 10.0)	(7.2, 9.6)	(7.4, 9.9)	(2.6, 8.8)	(0.8, 2.3)	(0.6, 7.3)	(0.3, 4.6)
p-value		0.19		0.68		0.0024		0.25
% change		-11.1%		3.2%		-71.1%		-48.3%
**Anatabine (ANTT)**								
Mean	17.0	14.5	13.7	13.7	7.2	1.2*†	3.1†	1.1†
(95% CI)	(14.0, 20.7)	(12.0, 17.5)	(11.5, 16.4)	(11.4, 16.4)	(3.3, 15.9)	(0.7, 2.2)	(0.6, 17)	(0.2, 6.7)
p-value		0.080		0.83		0.0006		0.19
% change		-14.8%		-0.7%		-83.1%		-64.0%
				**Tobacco Specific Nitrosamines (TSNAs) (ng/g creatinine)**				
**4-methylnitrosamino)-4-(3-pyridyl)-1-butanol (NNAL)**								
Mean	281.0	277.6	258.3	241.7	151.0	7.0*	40.2	3.8*
(95% CI)	(234.2, 337.2)	(231.1, 333.4)	(222.6, 299.7)	(208.0, 280.9)	(87.4, 260.8)	(3.7, 13.1)	(16.5, 97.9)	(2.2, 6.7)
p-value		0.56		0.25		<.0001		<.0001
% change		-1.2%		-6.4%		-95.4%		-90.5%
**N'-Nitrosonornicotine (NNN)**								
Mean	13.2	12.1	13.0	13.0	9.5	7.1	6.4	2.5*
(95% CI)	(10.9, 15.9)	(10.5, 14.0)	(10.9, 15.5)	(11.3, 15.0)	(6.7, 13.6)	(4.1, 12.3)	(3.4, 12.1)	(1.9, 3.4)
p-value		0.64		0.68		0.052		0.0005
% change		-8.3%		0.0%		-25.1%		-60.5%
**N'-nitrosoanatabine (NAT)**								
Mean	151.2	132.4	131.4	118.3	69.3	5.4*	28.3	4.2*
(95% CI)	(123.0, 185.8)	(109.2, 160.5)	(109.0, 158.4)	(97.9, 142.9)	(31.1, 154.6)	(3.7, 7.9)	(11.9, 67.4)	(3.0, 5.9)
p-value		0.31		0.16		<.0001		<.0001
% change		-12.4%		-10.0%		-92.2%		-85.2%
**N'-nitrosoanabasine (NAB)**								
Mean	23.6	19.6*	22.1	19.1*	13.2	1.8*†	5.8	1.4*†

	Exclusive Cigarette Smoking (N = 273)		Dual Cigarette/ENDS Use (N = 242)		Exclusive ENDS Use (N = 30)†		No Tobacco Use (N = 31)†	
Biomarker	Wave 1	Wave 2	Wave 1	Wave 2	Wave 1	Wave 2	Wave 1	Wave 2
(95% CI)	(19.6, 28.5)	(16.5, 23.4)	(19.0, 25.7)	(16.5, 22.1)	(7.1, 24.5)	(1.2, 2.7)	(2.9, 11.6)	(1.1, 1.9)
p-value		0.020		0.030		<.0001		<.0001
% change		-17.0%		-13.5%		-86.5%		-75.1%
**Heavy Metals (ug/g creatinine)**								
**Beryllium**								
Mean	0.01	0.01	0.01	0.01	0.01	0.02	0.01	0.01
(95% CI)	(0.01, 0.02)	(0.01, 0.01)	(0.01, 0.01)	(0.01, 0.01)	(0.01, 0.02)	(0.01, 0.03)	(0.01, 0.02)	(0.01, 0.02)
p-value		0.80		0.57		0.36		0.32
% change		0.0%		0.0%		100.0%		0.0%
**Cadmium**								
Mean	0.28	0.31*	0.28	0.31	0.29	0.35	0.16	0.16
(95% CI)	(0.25, 0.32)	(0.28, 0.35)	(0.24, 0.32)	(0.27, 0.35)	(0.20, 0.44)	(0.26, 0.48)	(0.11, 0.22)	(0.11, 0.21)
p-value		0.0088		0.10		0.55		0.87
% change		10.7%		10.7%		20.7%		0.0%
**Cobalt**								
Mean	0.57	0.56	0.60	0.60	0.82†	0.55*	0.57	0.48*
(95% CI)	(0.52, 0.62)	(0.50, 0.61)	(0.55, 0.66)	(0.54, 0.67)	(0.58, 1.15)	(0.43, 0.7)	(0.49, 0.66)	(0.42, 0.56)
p-value		0.96		0.82		0.0007		0.024
% change		-1.8%		0.0%		-32.9%		-15.8%
**Lead**								
Mean	0.50	0.46	0.49	0.50	0.61	0.46*	0.49	0.44
(95% CI)	(0.45, 0.55)	(0.42, 0.51)	(0.44, 0.53)	(0.45, 0.55)	(0.48, 0.77)	(0.35, 0.61)	(0.37, 0.64)	(0.32, 0.60)
p-value		0.22		0.43		0.0023		0.20
% change		-8.0%		2.0%		-24.6%		-10.2%
**Manganese**								
Mean	0.15	0.18	0.17	0.17	0.18	0.36†	0.13	0.11*
(95% CI)	(0.13, 0.16)	(0.13, 0.26)	(0.14, 0.20)	(0.15, 0.20)	(0.13, 0.25)	(0.17, 0.78)	(0.10, 0.19)	(0.09, 0.14)
p-value		0.13		0.99		0.065		0.0031
% change		20.0%		0.0%		100.0%		-15.4%
**Strontium**								
Mean	128.07	110.11	134.46	127.96	142.52	134.81	172.97	133.10
(95% CI)	(115.18, 142.40)	(93.69, 129.41)	(122.45, 147.64)	(117.16, 139.75)	(94.26, 215.49)	(94.33, 192.66)	(137.34, 217.85)	(89.98, 196.88)
p-value		0.14		0.25		0.42		0.051
% change		-14.0%		-4.8%		-5.4%		-23.1%

	Exclusive Cigarette Smoking (N = 273)		Dual Cigarette/ENDS Use (N = 242)		Exclusive ENDS Use (N = 30)†		No Tobacco Use (N = 31)†	
Biomarker	Wave 1	Wave 2	Wave 1	Wave 2	Wave 1	Wave 2	Wave 1	Wave 2
**Thallium**								
Mean	0.16	0.15	0.18	0.17	0.18	0.16	0.18	0.19
(95% CI)	(0.15, 0.17)	(0.14, 0.16)	(0.16, 0.19)	(0.16, 0.18)	(0.15, 0.21)	(0.13, 0.21)	(0.15, 0.21)	(0.16, 0.23)
p-value		0.94		0.57		0.22		0.59
% change		-6.3%		-5.6%		-11.1%		5.6%
**Uranium**								
Mean	0.01	0.01	0.01	0.01*	0.01	0.01	0.01	0.01
(95% CI)	(0.01, 0.01)	(0.01, 0.01)	(0.01, 0.01)	(0.01, 0.01)	(0.00, 0.01)	(0.01, 0.01)	(0.00, 0.01)	(0.00, 0.01)
p-value		0.73		0.0020		0.82		0.33
% change		0.0%		0.0%		0.0%		0.0%
**Arsenic and Arsenic Compounds (ug/g creatinine)**								
**Total Inorganic Arsenic**								
Mean	4.8	4.2	4.6	4.4	5.4	4.8*	4.7	4.6*
(95% CI)	(4.4, 5.3)	(3.8, 4.5)	(4.2, 5.0)	(4.0, 4.8)	(4.0, 7.3)	(3.6, 6.2)	(3.4, 6.5)	(3.5, 6.0)
p-value		0.011		0.29		0.037		0.51
% change		-12.5%		-4.3%		-11.1%		2.1%
**Polycyclic Aromatic Hydrocarbons (ng/g creatinine)§**								
**1-Naphthol or 1-hydroxynaphthalene (1-NAP) (ug/g)**								
Mean	14.4	12.5	12.7	12.8	6.6	2.4†	9.9†	4.0*†
(95% CI)	(12.0, 17.4)	(10.6, 14.6)	(10.7, 15.1)	(10.2, 16.1)	(3.7, 12)	(0.5, 12.8)	(2.5, 39)	(0.9, 16.8)
p-value		0.067		0.96		0.25		0.016
% change		-13.6%		0.6%		-63.1%		-60.0%
**2-Naphthol or 2-hydroxynaphthalene (2-NAP) (ug/g)**								
Mean	15.8	15.7	13.9	14.6	11.7	5.5*	8.0	5.0
(95% CI)	(14.7, 16.9)	(14.6, 16.9)	(12.9, 15)	(13, 16.3)	(7.7, 17.8)	(3.9, 7.7)	(5.5, 11.5)	(3.6, 7.0)
p-value		0.73		0.39		0.0048		0.047
% change		-0.4%		4.5%		-52.7%		-37.2%
**3-Hydroxyfluorene (3-FLU)**								
Mean	675.7	663.1	614.7	627.1	488.0	91.5*	215.9	92.4*
(95% CI)	(607.2, 752.0)	(582.7, 754.5)	(556.0, 679.6)	(562.9, 698.8)	(320.1, 744.0)	(53.9, 155.1)	(127.2, 366.6)	(68.2, 125.1)
p-value		0.98		0.71		<.0001		0.0020
% change		-1.9%		2.0%		-81.3%		-57.2%
**2-Hydroxyfluorene (2-FLU)**								

Biomarker	Exclusive Cigarette Smoking (N = 273) Wave 1	Exclusive Cigarette Smoking (N = 273) Wave 2	Dual Cigarette/ENDS Use (N = 242) Wave 1	Dual Cigarette/ENDS Use (N = 242) Wave 2	Exclusive ENDS Use (N = 30)† Wave 1	Exclusive ENDS Use (N = 30)† Wave 2	No Tobacco Use (N = 31)† Wave 1	No Tobacco Use (N = 31)† Wave 2
Mean	1208.0	1163.1	1098.0	1111.8	915.6	229.1*	470.2	211.3*
(95% CI)	(1096.1, 1331.4)	(1053.4, 1284.2)	(1001.7, 1203.6)	(1003.4, 1231.8)	(644.7, 1300.4)	(145.9, 359.8)	(292.1, 756.8)	(157.1, 284.2)
p-value		0.60		0.82		<.0001		0.0015
% change		-3.7%		1.3%		-75.0%		-55.1%
**1-Hydroxyphenanthrene (1-PHE)**								
Mean	203.0	214.5*	193.6	211.6*	180.6	154.5	173.7	138.9
(95% CI)	(189.1, 217.9)	(198.3, 231.9)	(180.1, 208.1)	(195.5, 229.0)	(135.4, 240.8)	(101.5, 235.3)	(126.3, 238.8)	(117.2, 164.7)
p-value		0.012		0.012		0.21		0.19
% change		5.6%		9.3%		-14.4%		-20.0%
**1-Hydroxypyrene (1-PYR)**								
Mean	371.8	352.6	338.1	342.5	295.1	151.7*	211.3	139.7*
(95% CI)	(341.5, 404.9)	(319.7, 388.9)	(311.6, 366.7)	(309.3, 379.2)	(234.0, 372.2)	(102.4, 224.8)	(143.1, 312.2)	(115.8, 168.5)
p-value		0.27		0.86		0.0001		0.013
% change		-5.2%		1.3%		-48.6%		-33.9%
**Sum of 2-Hydroxyphenanthrene and 3-Hydroxyphenanthrene (2-3PHE)**								
Mean	325.0	317.3	293.6	309.6	290.3	169.1*	205.4	134.6*
(95% CI)	(297.9, 354.6)	(288.6, 348.7)	(272.9, 315.8)	(281.8, 340.2)	(222.2, 379.1)	(110.4, 259.0)	(137.2, 307.4)	(109.5, 165.4)
p-value		0.82		0.21		0.0010		0.041
% change		-2.4%		5.4%		-41.7%		-34.5%
**Volatile Organic Compound Metabolites (VOCM) (ug/g creatinine)**								
**3- & 4-Methylhippuric acid (3MHA/4MHA) (Xylene)**								
Mean	122.3	119.9	109.5	131.9	94.8	32.9	53.0	33.4
(95% CI)	(108.3, 138.2)	(107.1, 134.3)	(97.1, 123.5)	(114.8, 151.5)	(59.5, 150.9)	(23.3, 46.6)	(33.1, 84.7)	(24.2, 45.9)
p-value		0.86		0.0008		0.0003		0.033
% change		-2.0%		20.4%		-65.2%		-37.0%
**3, 4-Methylhippuric acid (34MH) (Xylene)**								
Mean	826.2	743.1	716.5	755.0	645.5	141.8*	358.3	210.4*
(95% CI)	(733.8, 930.3)	(674.9, 818.2)	(641.7, 799.9)	(669.4, 851.5)	(482.2, 864.3)	(95.1, 211.3)	(231.1, 555.5)	(147.1, 301.0)
p-value		0.10		0.28		<.0001		0.021
% change		-10.1%		5.4%		-78.0%		-41.3%
**N-Acetyl-S-(2-carbamoylethyl)-L-cysteine (CaEMA) (Acrylamide)**								
Mean	149.9	135.4	143.2	136.2	113.0	40.0*	84.1	53.8*
(95% CI)	(137.1, 163.9)	(125.8, 145.8)	(132.9, 154.4)	(127.0, 146.1)	(80.3, 159.1)	(31.6, 50.7)	(62.3, 113.6)	(39.8, 72.8)
p-value		0.028		0.17		<.0001		0.0019

Biomarker	Exclusive Cigarette Smoking (N = 273) Wave 1	Exclusive Cigarette Smoking (N = 273) Wave 2	Dual Cigarette/ENDS Use (N = 242) Wave 1	Dual Cigarette/ENDS Use (N = 242) Wave 2	Exclusive ENDS Use (N = 30)† Wave 1	Exclusive ENDS Use (N = 30)† Wave 2	No Tobacco Use (N = 31)† Wave 1	No Tobacco Use (N = 31)† Wave 2
% change		-9.7%		-4.9%		-64.6%		-36.0%
**N-Acetyl-S-(Nmethylcarbamoyl)-L-cysteine (MCaMA) (N,N-Dimethylformamide/isocyanates)**								
Mean	568.7	569.4	545.5	555.7	381.9	195.0*	274.8	173.9*
(95% CI)	(515.7, 627.1)	(516.8, 627.4)	(493.0, 603.6)	(505.7, 610.8)	(274.3, 531.8)	(137.2, 277.2)	(181.6, 416.0)	(140.3, 215.6)
p-value		0.31		0.65		0.0012		0.013
% change		0.1%		1.9%		-48.9%		-36.7%
**N-Acetyl-S-(benzyl)-L-cysteine (BzMA) (Toluene)**								
Mean	6.9	6.2	6.8	6.7	4.7	6.0*	7.7	7.9
(95% CI)	(6.1, 7.8)	(5.5, 7.1)	(5.9, 7.8)	(5.9, 7.6)	(3.5, 6.2)	(4.7, 7.7)	(5.9, 10.1)	(5.7, 10.9)
p-value		0.13		0.85		0.14		0.92
% change		-9.6%		-1.0%		29.7%		2.5%
**N-Acetyl-S-(2-carboxyethyl)-L-cysteine (2CoEMA) (Acrolein)**								
Mean	326.8	301.4	299.6	303.8	245.2	104.8*	152.6	97.8
(95% CI)	(293.4, 364.0)	(271.0, 335.2)	(275.4, 325.9)	(278.5, 331.4)	(171.7, 350.3)	(71.1, 154.5)	(110.0, 211.7)	(74.6, 128.3)
p-value		0.16		0.81		0.0003		0.0025
% change		-7.8%		1.4%		-57.3%		-35.9%
**N-Acetyl-S-(1-cyano-2-hydroxyethyl)-L-cysteine (1CyHEMA) (Acrylonitrile)**								
Mean	28.9	27.3	26.4	27.6	17.2	3.2*	9.0	3.2*
(95% CI)	(23.9, 35.0)	(22.8, 32.6)	(22.9, 30.3)	(24.1, 31.7)	(10.0, 29.8)	(2.2, 4.8)	(4.8, 16.7)	(2.2, 4.6)
p-value		0.58		0.48		<.0001		0.0019
% change		-5.7%		4.8%		-81.3%		-64.8%
**N-Acetyl-S-(2-cyanoethyl)-L-cysteine (2CyEMA) (Acrylonitrile)**								
Mean	162.1	149.8	141.2	136.4	72.7	2.8*	27.1	3.9*
(95% CI)	(137.3, 191.4)	(129.7, 173.1)	(122.7, 162.4)	(120.2, 154.7)	(33.9, 156.1)	(1.7, 4.6)	(10.8, 68.1)	(1.9, 7.7)
p-value		0.40		0.56		<.0001		<.0001
% change		-7.6%		-3.4%		-96.2%		-85.8%
**N-Acetyl-S-(3,4-dihydroxybutyl)-L-cysteine (34HBMA) (1,3 Butadiene)**								
Mean	543.7	516.8	530.1	534.5*	452.1	338.6*	440.3	397.5*
(95% CI)	(512.7, 576.5)	(483.0, 552.9)	(504.1, 557.5)	(505.2, 565.5)	(380.7, 536.9)	(299.4, 383.1)	(377.3, 513.8)	(349.8, 451.7)
p-value		0.18		0.92		0.0007		0.20
% change		-4.9%		0.8%		-25.1%		-9.7%
**N-Acetyl-S-(2-carbamoyl-2-hydroxyethyl)-L-cysteine (2CaHEMA) (Acrylamide)**								
Mean	18.7	17.9	19.2	19.3	17.8	14.4*	13.3	11.4*
(95% CI)	(17.3, 20.2)	(16.4, 19.6)	(17.7, 20.8)	(17.7, 21.0)	(14.7, 21.6)	(10.7, 19.4)	(10.2, 17.3)	(8.9, 14.5)
p-value		0.53		0.89		0.0058		0.18

	Exclusive Cigarette Smoking (N = 273)		Dual Cigarette/ENDS Use (N = 242)		Exclusive ENDS Use (N = 30)†		No Tobacco Use (N = 31)†	
Biomarker	Wave 1	Wave 2	Wave 1	Wave 2	Wave 1	Wave 2	Wave 1	Wave 2
% change		-4.2%		0.3%		-19.1%		-14.4%
**N-Acetyl-S-(2-hydroxyethyl)-L-cysteine (2HEMA) (Acrylonitrile, vinyl chloride, ethylene oxide)**								
Mean	3.2	3.3	3.6	3.6	2.8	1.2*†	1.7†	1.0*†
(95% CI)	(2.7, 3.9)	(2.9, 3.7)	(3.1, 4.1)	(3.1, 4.2)	(2.1, 3.8)	(0.9, 1.7)	(1.0, 2.8)	(0.8, 1.3)
p-value		0.40		0.87		0.0002		0.0014
% change		0.6%		1.4%		-57.1%		-42.6%
**N-Acetyl-S-(2-hydroxypropyl)-L-cysteine (2HPMA) (Propylene Oxide)**								
Mean	87.8	79.2	84.8	78.0	81.7	27.3*	58.8	36.7
(95% CI)	(75.8, 101.8)	(68.8, 91.2)	(74.0, 97.2)	(69.7, 87.2)	(54.2, 123.2)	(21.5, 34.6)	(37.6, 91.9)	(26.9, 50.0)
p-value		0.14		0.19		0.0002		0.0074
% change		-9.8%		-8.1%		-66.6%		-37.5%
**N-Acetyl-S-(3-hydroxypropyl)-L-cysteine (3HPMA) (Acrolein)**								
Mean	1418.2	1450.6	1294.9	1447.2	915.4	306.5*	664.5	363.9*
(95% CI)	(1225.1, 1641.8)	(1274.9, 1650.5)	(1163.0, 1441.7)	(1291.3, 1622.0)	(659.7, 1270.2)	(231.0, 406.6)	(431.0, 1024.4)	(260.1, 509.1)
p-value		0.23		0.046		<.0001		0.013
% change		2.3%		11.8%		-66.5%		-45.2%
**N-Acetyl-S-(3-hydroxypropyl-1-methyl)-L-cysteine (3HMPMA) (Crotonaldehyde)**								
Mean	1698.5	1542.1	1551.3	1522.3	879.8	227.7	645.2	301.2
(95% CI)	(1505.4, 1916.5)	(1368.1, 1738.2)	(1398.5, 1720.7)	(1357.2, 1707.5)	(551.8, 1402.6)	(183.2, 283.0)	(337.2, 1234.4)	(236.4, 383.6)
p-value		0.15		0.71		<.0001		0.0082
% change		-9.2%		-1.8%		-74.1%		-53.3%
**N-Acetyl-S-(4-hydroxy-2-methyl-2-buten-1-yl)-L-cysteine (4HMBeMA) (Isoprene)**								
Mean	45.3	41.8	40.2	42.3	24.7	3.5	14.8	7.0
(95% CI)	(38.9, 52.7)	(36.6, 47.8)	(34.9, 46.4)	(36.1, 49.5)	(16.4, 37.4)	(2.9, 4.2)	(7.4, 29.6)	(4.4, 11.4)
p-value		0.43		0.53		<.0001		0.0023
% change		-7.6%		5.1%		-85.8%		-52.5%
**Mandelic acid (MADA)**								
Mean	312.8	299.7*	282.2	297.2*	244.7	116.3	198.5	159.2
(95% CI)	(282.5, 346.4)	(274.8, 326.8)	(261.5, 304.5)	(273.2, 323.5)	(178.6, 335.2)	(76.7, 176.4)	(148.2, 265.9)	(131.6, 192.7)
p-value		0.41		0.26		0.63		0.049
% change		-4.2%		5.3%		-52.5%		-19.8%
**N-Acetyl-S-(4-hydroxy-2-buten-1-yl)-L-cysteine (4HBeMA) (1,3 Butadiene)**								
Mean	35.4	33.9	31.9	33.8	18.8	2.8*	13.0	5.6*
(95% CI)	(30.4, 41.3)	(29.9, 38.4)	(28.1, 36.1)	(29.9, 38.3)	(10.8, 32.9)	(2.2, 3.7)	(7.0, 24.1)	(4.2, 7.4)
p-value		0.77		0.34		<.0001		0.0052

Biomarker	Exclusive Cigarette Smoking (N = 273) Wave 1	Exclusive Cigarette Smoking (N = 273) Wave 2	Dual Cigarette/ENDS Use (N = 242) Wave 1	Dual Cigarette/ENDS Use (N = 242) Wave 2	Exclusive ENDS Use (N = 30)† Wave 1	Exclusive ENDS Use (N = 30)† Wave 2	No Tobacco Use (N = 31)† Wave 1	No Tobacco Use (N = 31)† Wave 2
% change		-4.3%		6.2%		-85.0%		-57.2%
**Phenylglyoxylic acid (PhGA) (Ethylbenzene, styrene)**								
Mean	438.0	422.1	393.5	457.7*	396.8	240.7*	271.7	259.7
(95% CI)	(400.7, 478.7)	(381.6, 466.9)	(355.2, 436.0)	(426.9, 490.7)	(318.2, 494.8)	(202.6, 285.9)	(226.6, 325.8)	(221.5, 304.4)
p-value		0.70		0.0038		<.0001		0.59
% change		-3.6%		16.3%		-39.3%		-4.4%
**N-Acetyl-S-(phenyl)-L-cysteine (PhMA) (Benzene)**								
Mean	1.1†	0.9†	1.1†	1.1†	1.0†	1.1†	1.1†	1.0†
(95% CI)	(1.0, 1.2)	(0.8, 1.1)	(1.0, 1.2)	(1.0, 1.2)	(0.7, 1.4)	(1.0, 1.3)	(0.8, 1.5)	(0.7, 1.4)
p-value		0.13		0.99		0.87		0.38
% change		-13.2%		0.0%		10.8%		-9.2%
**2-Thioxothiazolidine-4-carboxylic acid (TTCA) (Carbon Disulfide)**								
Mean	22.9	15.0*	23.8	19.1*	20.0	18.8	18.7	23.4
(95% CI)	(20.0, 26.3)	(13.6, 16.6)	(21.0, 27.1)	(16.1, 22.6)	(13.6, 29.4)	(12.6, 28.3)	(12.6, 27.9)	(13.9, 39.5)
p-value		<.0001		0.007		0.49		0.49
% change		-34.7%		-19.9%		-5.9%		25.0%

Abbreviations: PATH – Population Assessment of Tobacco and Health, ENDS – electronic nicotine delivery system, CI – confidence interval. All estimates are weighted.

All geometric means are creatinine adjusted. P-values are for the Wave 1 to Wave 2 change in biomarker levels. % change indicates the Wave 1 to Wave 2 change in geometric mean by Wave 2 tobacco product use group.

* Indicates a statistically significant change (p < 0.05) in biomarker levels from Wave 1 to Wave 2.

† Estimate should be interpreted with caution because it has low statistical precision. The coefficient of variation of the estimate is larger than 30% or the sample size is <50.

§Concentrations of 1-NAP and 2-NAP are ug/g, all other polycyclic aromatic hydrocarbon biomarker concentrations are in ng/g.
